# Echovirus 11 infection induces pyroptotic cell death by facilitating NLRP3 inflammasome activation

**DOI:** 10.1371/journal.ppat.1010787

**Published:** 2022-08-26

**Authors:** Chong Wang, Ruyi Yang, Fengxia Yang, Yang Han, Yujie Ren, Xiaobei Xiong, Xingyun Wang, Yidan Bi, Lijun Li, Yang Qiu, Yi Xu, Xi Zhou

**Affiliations:** 1 Guangzhou Institute of Pediatrics, Guangzhou Women and Children’s Medical Center, Guangzhou, Guangdong, China; 2 State Key Laboratory of Virology, Wuhan Institute of Virology, Chinese Academy of Sciences, Wuhan, Hubei, China; 3 Joint Laboratory of Infectious Diseases and Health, Wuhan Institute of Virology & Wuhan Jinyintan Hospital, Wuhan Jinyintan Hospital, Wuhan, Hubei, China; Chang Gung University, TAIWAN

## Abstract

Echovirus 11 (ECHO 11) is a positive-strand RNA virus belonging to the genus *Enterovirus* of the family *Picornaviridae*. ECHO 11 infections can cause severe inflammatory illnesses in neonates, including severe acute hepatitis with coagulopathy. The activation of NLRP3 inflammasome is important for host defense against invading viruses, which also contributes to viral pathogenicity. However, whether and how ECHO 11 induces NLRP3 inflammasome activation remains unclear. In this study, we isolated a clinical strain of ECHO 11 from stools of an ECHO 11-infected newborn patient with necrotizing hepatitis. This virus shared 99.95% sequence identity with the previously published ECHO 11 sequence. The clinically isolated ECHO 11 can efficiently infect liver cells and strongly induces inflammation. Moreover, we showed that ECHO 11 induced IL-1β secretion and pyroptosis in cells and mouse bone marrow-derived macrophages (BMDMs). Furthermore, ECHO 11 infection triggered NLRP3 inflammasome activation, as evidenced by cleavages of GSDMD, pro-IL-1β and pro-caspase-1, and the release of LDH. ECHO 11 2B protein was required for NLRP3 inflammasome activation via interacting with NLRP3 to facilitate the inflammasome complex assembly. *In vivo*, expression of ECHO 11 2B also activated NLRP3 inflammasome in the murine liver. Besides, 2Bs of multiple EVs can also interact with NLRP3 and induce NLRP3 inflammasome activation. Together, our findings demonstrate a mechanism by which ECHO 11 induces inflammatory responses by activating NLRP3 inflammasome, providing novel insights into the pathogenesis of ECHO 11 infection.

## Author summary

NLRP3 inflammasome is important for host defense against invading viruses, and contributes to viral pathogenicity. Human echovirus 11 (ECHO 11) belongs to the *Enterovirus* genus from the family *Picornavirida*, and it can cause severe acute hepatitis in newborns with high morbidity and mortality. However, the knowledge about the pathogenesis of ECHO 11 infection is limited. Whether and how ECHO 11 induces NLRP3 inflammasome activation remains unclear. This work provides the first demonstration that ECHO 11 can induce inflammatory responses via activating NLRP3 inflammasome and pyroptosis. More importantly, ECHO 11-encoded 2B protein was found to activate NLRP3 inflammasome in cells and *in vivo*, and the interaction between 2B and NLRP3 was required for inflammasome complex assembly. Furthermore, we uncovered that 2Bs of other enteroviruses, including enterovirus 71, coxsackievirus A16 (CVA16) and CVB3 could induce NLRP3 inflammasome and interact with NLRP3. Our findings uncover a mechanism by which ECHO 11 induces inflammatory responses and demonstrate a novel function of ECHO 11 2B.

## Introduction

Enteroviruses (EVs) are a large group of positive-sense single-stranded RNA viruses belonging to the *Enterovirus* genus from the family *Picornaviridae*, and include four human enterovirus species, EV-A to–D [1]. Among them, human echovirus 11 (ECHO 11) belongs to EV-B and is one of the most commonly isolated enteroviruses [2–4]. Because it can cause only a cytopathic effect with no pathogenicity to experimental animals, it is named as an “enteric cytopathogenic human orphan virus”. ECHO 11 infections are associated with a broad spectrum of illnesses, ranging from mild nonspecific symptoms to systemic disorders, including aseptic meningitis, upper respiratory tract infections, myocarditis and neonatal hepatitis [5–7]. The outbreak of ECHO 11 frequently occurs, and it has been reported to cause severe conditions, particularly severe acute hepatitis, in newborns with high morbidity and mortality, causing great social panic [4,8]. However, despite its important impact on human health, the mechanism about the pathogenesis of ECHO 11 infection is barely understood, which hinders the development of effective therapeutic strategies.

Host innate immune system directly senses the viral components by pattern recognition receptors (PRRs) and elicits inflammatory responses by activating inflammasomes to establish the anti-viral status [9]. Inflammasomes consist of a PRR as the viral sensor, an adaptor protein that usually is an apoptosis-associated speck-like protein containing a caspase-recruitment domain (ASC), and the cysteine protease caspase-1 (CASP-1) [10]. The nucleotide-binding domain and leucine-rich repeat containing receptor (NLR) family PYRIN domain containing-3 (NLRP3) inflammasome is one of the best-characterized inflammasomes. Upon activation, NLRP3 inflammasome operates as a platform for CASP-1 activation, resulting in CASP-1-dependent proteolytic maturation and cleavage of pro-interleukin-1β (IL-1β) [11]. The activated caspase-1 cleaves the pore-forming protein gasdermin D (GSDMD), which disrupts the cellular plasma membrane integrity, enabling the secretion of mature IL-1β, and inducing inflammatory program cell death termed pyroptosis [12–14]. This, in turn, activates and exacerbates the immune and inflammation responses and facilitates the recruitment of immune cells to the infection sites, controlling the invading viruses. The virus-activated NLRP3 inflammasome and pyroptosis can induce immune/inflammatory response and inhibit viral replication, while excessive and prolonged inflammation can also cause severe immunopathology, which is tightly linked to the viral pathogenicity and clinical outcomes [15,16].

The activation of NLRP3 inflammasome and pyroptosis is an important part of innate immune response during the infections of EVs. Previous studies on human enterovirus 71 (EV71), belonging to EV-A, have shown that its infection induces the production and secretion of IL-1β in THP1 cells, macrophages, and peripheral blood mononuclear cells (PBMCs), and mouse bone marrow-derived macrophages (BMDMs) [17]. The NLRP3 inflammasome plays protective role against the infections of EV71 and coxsackievirus B3 (CVB3) that is another member of EV-B, as evidenced by that the higher viral load and severe symptoms were observed in the NLRP3 inflammasome-deficient mice [18,19]. Moreover, recent genetic study has shown that EV71 3D can directly activate NLRP3 inflammasome via facilitating NLRP3 inflammasome assembly [17]. However, it remains unknown whether and how ECHO 11 triggers NLRP3 inflammasome and pyroptosis.

In this study, we isolated an ECHO 11 strain from a clinical specimen from a newborn patient with confirmed ECHO 11 infection at Guangzhou Women and Children′s Medical Center, China. We found that this ECHO 11 can efficiently infect liver cells and induce inflammation. ECHO 11 infection triggered NLRP3 inflammasome activation and pyroptosis in THP-1 and mice BMDMs. Moreover, ECHO 11-encoded 2B protein interacted with NLRP3 to facilitate the complex assembly of NLRP3 inflammasome. We confirmed that 2B also activated NLRP3 inflammasome *in vivo*. Furthermore, we uncovered that 2Bs of other EVs, including EV71, CVA16 and CVB3 can interact with NLRP3 and induce pyroptosis depended on NLRP3 inflammasome. Our findings uncover a mechanism by which ECHO 11 induces inflammatory responses and demonstrate a novel function of ECHO 11 2B.

## Results

### Fatal cases caused by ECHO 11 are associated with fulminant hepatic failure

In June 2019, six ECHO 11-infected newborns were transferred to the neonatal intensive care unit of Guangzhou Women and Children′s Medical Center, China. Among them, two patients (A and B) unfortunately passed away due to ECHO 11-associated necrotizing hepatitis. ECHO 11 viral RNA was detected via qRT-PCR in throat and nasal swab samples from Patients A and B (Table 1). We excluded the potential infections by other pathogens via examining the serum and swab samples from the patients. The dynamic laboratory testing indicated significantly increased levels of coagulation and liver indices, such as direct bilirubin (DBIL), indirect bilirubin (IBIL), Alanine aminotransferase (ALT), Aspartate aminotransferase (AST), etc. These markers were maintained at a high level until the death of the patients. The platelet counts decreased along with prothrombin time (PT) and activated partial thromboplastin time (APTT). Cytokine expressions were remarkably increased. Hepatic encephalopathy was confirmed with the presence of increased blood ammonia in both patients. All these data showed that both patients were suffering acute hepatic failure and renal failure with severe coagulation disturbance, myocardial damage and pancreatitis. Moreover, liver tissues of both patients were collected via postmortem needle puncture, and ECHO 11 viral RNA was detected, suggesting that ECHO 11 can directly infect the liver and cause severe damage (Table 1).

**Table 1:** Characteristics of newborn patients with ECHO 11.

	Patient A			Patient B		
Postnatal days	8	15	18	8	15	18
Throat and nasal swab for ECHO 11	Positive	—	Positive	Positive	Positive	Positive
Postmortem liver for ECHO 11			Positive			
**Co-infection**						
Other viruses	ND			ND		
Bacteria	ND			ND		
Fungus	ND			ND		
**Blood routine**						
WBC (×10^9^/L, normal range 4–10)	13.5	1.6	2.3	9.5	3.6	3.4
Neutrophils (×10^9^/L, normal range 2–8)	5.26	1.22	1.91	3.44	2.06	1.64
Lymphocyte (×10^9^/L, normal range 1.1–3.2)	6.39	0.09	0.13	3.11	1.13	1.15
PLT (×10^9^/L, normal range 100–300)	21	50	34	7	17	23
HGB (×10^9^/L, normal range 170–200)	31	69	82	37	64	134
CRP (mg/L, normal range 0–10)	2.1	2.1	—	4.5	9.3	4.59
APTT (s, normal range 25.1–36.5)	142.5	93.4	72.6	180	78.3	56.6
PT (s, normal range 9.4–12.5)	34.9	17.6	16.3	98.1	26.3	15.9
FIB (g/L, normal range 2–4)	0.72	2.69	2.91	<0.6	1.06	2.25
Lac (mmoL/L, normal range 0.5–1.7)	3.7	14.7	5.9	4.5	11.9	8.7
GLU(mmoL/L, normal range 3.9–6.1)	2.6	4.6	3.46	1.3	7.7	4.4
ALT (U/L, normal range 9–50)	228 ↑	17	24	285 ↑	20	21
AST (U/L, normal range 15–40)	3055 ↑	115↑	108 ↑	3255 ↑	197 ↑	103 ↑
ALP (U/L, normal range 50–125)	253 ↑	17↑	209 ↑	352 ↑	339 ↑	172 ↑
γ-GT (U/L, normal range 11–50)	206	28	37	145	43	87
TBIL (μmoL/L, normal range 0–18.8)	6.4	186.1	274	99	319.7	363.1
DBIL (μmoL/L, normal range 0–6.8)	20.2	25.4	36.3	43.3	87.2	67.3
IBIL (μmoL/L, normal range 1.7–10.2)	47.2	160.7	237.7	55.7	232.5	295.8
ALB (μmoL/L, normal range 38–42)	20.8	54.4	56.2	24.8	33.3	41.6
AMON (umol/L, normal range 18–72)	132.3	74.2	—	151	—	—
cTnI (μg/L, normal range 0–0.2)	Negative	—	0.61	—	1.43	—
PCT (ng/mL, normal range 0–0.05)	0.692	—	0.138	0.372	0.354	—
CD3+CD4+T (cells/μL, 706–1125)	—	—	118.65	—	—	231.55
CD3+CD8+T (cells/μL, 323–836)	—	—	30.06	—	—	67.12
**Cytokines**						
IL-6 (pg/mL, normal range 0–8.8)	—	—	4760.02↑	—	1939.25 ↑	—
IL-8 (pg/mL, normal range 0–15.7)	—	—	3074.33↑	—	2121.41 ↑	—
IL-1β (pg/mL, normal range 0–3.12)	—	—	4.55 ↑	—	2.54	—
IL-10 (pg/mL, normal range 0–8.41)	—	—	28.14 ↑	—	23.16 ↑	—
IL-17A (pg/mL, normal range 0–3.71)	—	—	12.62 ↑	—	8.74 ↑	—
TNFα (pg/mL, normal range 0–5.35)	—	—	26.65 ↑	—	11.42 ↑	—
IFN-γ (pg/mL, normal range 0–6.56)	—	—	8.23↑	—	3.33	—

Arrows (↑) indicate values beyond the normal range

### ECHO 11 efficiently infects liver cell lines and induces inflammation

**Fig 1:**
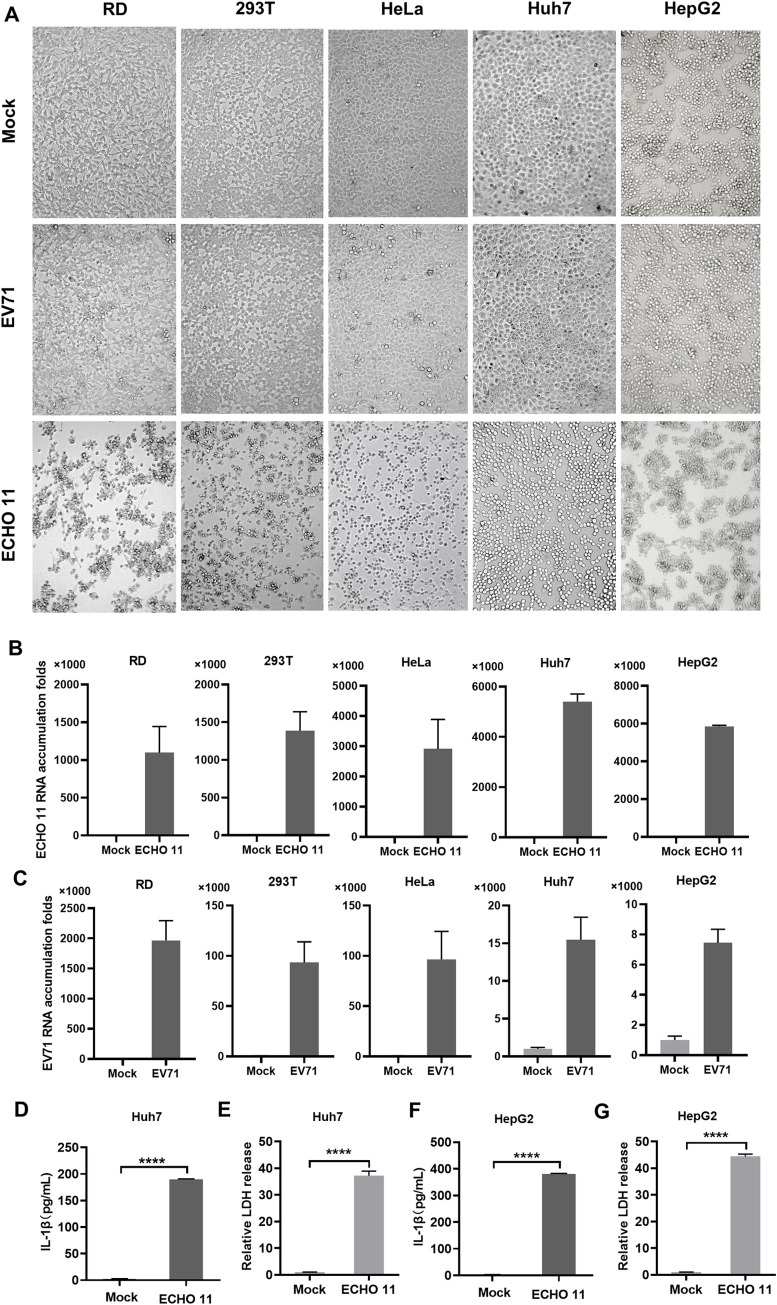
ECHO 11 efficiently infects liver cell lines and induces inflammation. (**A**) RD, 293T, HeLa, Huh7 and HepG2 cells were infected with EV71 or ECHO 11 at an MOI of 0.1, and at 24 h.p.i., CPE was examined by inverted microscope observations. (**B-C**) The accumulation of viral RNA in ECHO 11- and EV71- infected cells was measured by qRT-PCR at 24 h.p.i., and the level of viral RNA in each cell line without infection (Mock) was defined as 1-fold. (**D-G**) Huh7 or HepG2 cells were infected with ECHO 11 at an MOI of 0.1, and at 24 h.p.i., and mature IL-1β levels in the supernatants were determined by ELISA (**D**, **F**). The LDH release in the supernatants were determined by cytotoxicity assay, and the level of LDH in mock cells was defined as 1-fold (**E**, **G**). Data represent means and SD from three repeated experiments. *, *P* < 0.05; **, *P* < 0.01; ***, *P* < 0.001; ****, *P* < 0.0001, as measured by unpaired *t* test.

We successfully isolated the virus in human rhabdomyosarcoma (RD) cells using stool samples of Patient B (Fig 1A), and the virus was analyzed via RNA sequencing. A total of 4,918,963 obtained reads were used to align to a set of 43 Echovirus references with the complete genome. The results showed that this virus shared 99.95% sequence identity to Human Echovirus 11 strain D207 (EF634316.1), which was previously isolated from a stool specimen of a diabetic 12-year-old child in Slovakia [20]. We then named it Human echovirus 11 strain GWCMC01/GZ/CHN/2019, and submitted the sequence to NCBI (MN817130).

Because ECHO 11 viral RNA can be detected in the livers of patients, we sought to test whether it can directly infect liver cells. Thus, RD, 293T, HeLa, Huh7 and HepG2 cell lines were used to infect with this newly identified ECHO 11 strain (short for ECHO 11) at a multiplicity of infections (MOI) of 0.1, and the replicative efficiencies of ECHO 11 in different cells were determined via examining the viral RNA accumulation at 24 h post infection (h.p.i.) with qRT-PCR. We observed severe cytopathic effect (CPE) in these cells at 24 h.p.i. (Fig 1A). Moreover, our data showed that the liver cell lines, including Huh7 and HepG2, can support ECHO 11 infection (Fig 1B). As a control, EV71 (strain H) expectedly replicated efficiently on these cells (Fig 1C). Furthermore, we found that mature IL-1β secretion and the release of the cytosolic enzyme lactate dehydrogenase (LDH) were induced by ECHO 11 in both Huh7 and HepG2 cells (Fig 1D–1G). The mRNA levels of pro-inflammatory genes, including IL-1β, IL-6, IFN-α, IFN-β and IFN-γ, were significantly stimulated in ECHO 11-infected liver cells (S1A and S1B Fig). Therefore, our findings indicate that ECHO 11 can efficiently infect liver cells and strongly induce inflammation, consistent with the clinical data.

### ECHO 11 infection induces pyroptotic cell death

**Fig 2:**
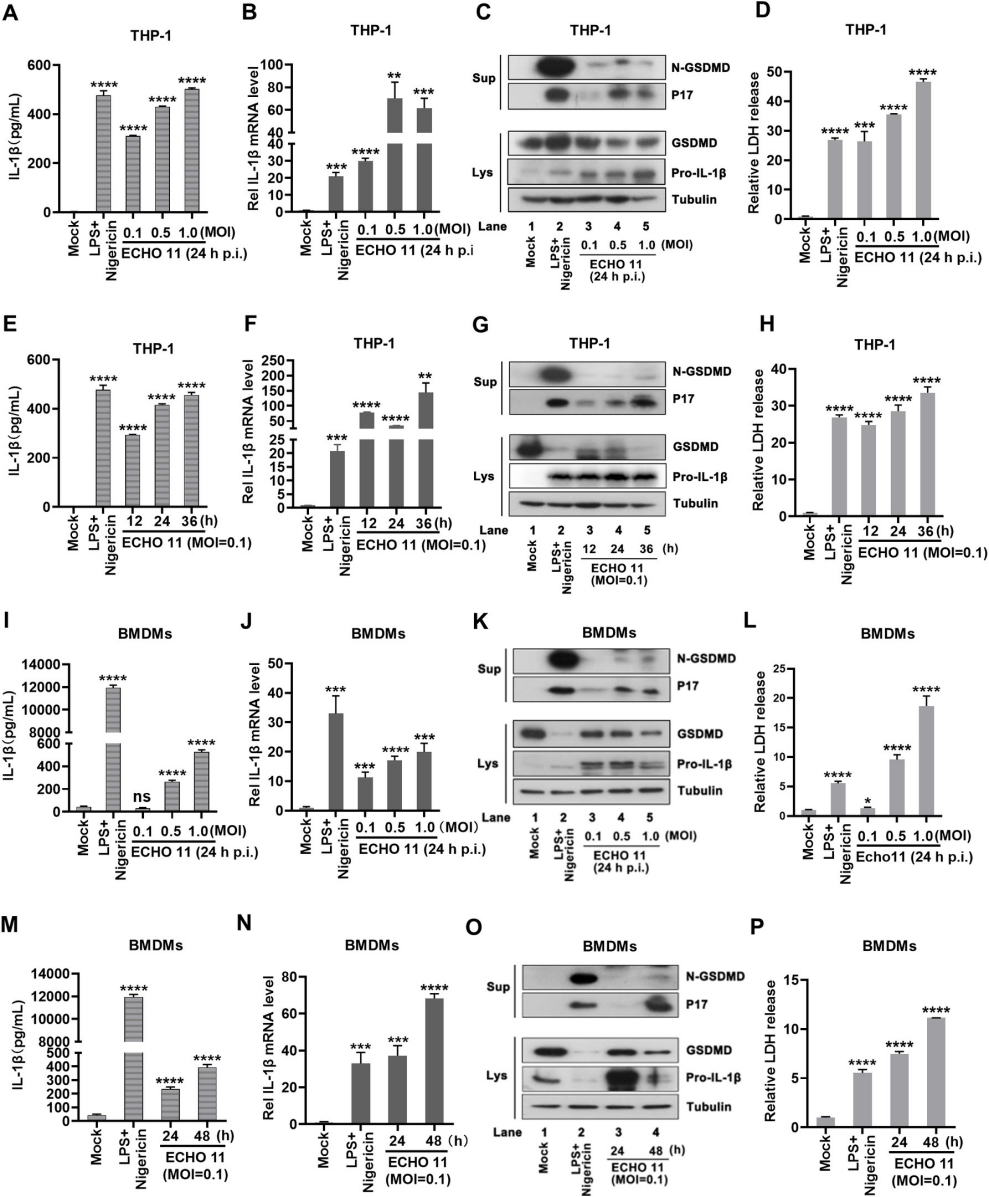
ECHO 11 infection induces pyroptosis in cells and *ex vivo*. THP-1 cells (**A-D**) or BMDMs (**I-L**) were infected with ECHO 11 at an MOI of 0.1, 0.5 or 1 for 24 h or THP-1 cells (**E-H**) or BMDMs (**M-P**) were infected with ECHO 11 at an MOI of 0.1 for 12, 24 or 36 h. Cells treated with LPS (10 μg/ml) for 5 h and 10 μM Nigericin for 1 h was used as the positive control, while cells without treatment (Mock) were used as the negative control. (**A**, **E**, **I** and **M**) Mature IL-1β levels in the supernatants were determined by ELISA. (**B**, **F**, **J** and **N**) The intracellular mRNA levels of IL-1β were measured by qRT-PCR, and the level of IL-1β mRNA in mock cells were defined as 1-fold. (**C**, **G**, **K** and **O**) GSDMD-N and P17 in the supernatants (Sup), and GSDMD and pro-IL-1β in cell lysates (Lys) were examined by Western blotting with the indicated antibodies. (**D**, **H**, **L** and **P**) The LDH release in the supernatants were determined by cytotoxicity assay, and the level of LDH in mock cells was defined as 1-fold. Data represent means and SD from three repeated experiments. *, *P* < 0.05; **, *P* < 0.01; ***, *P* < 0.001; ****, *P* < 0.0001, as measured by one-way ANOVA.

The observations showed that ECHO 11 infection induced IL-1β secretion and LDH release gave us a hint that ECHO 11 infection may induce inflammasome activation and pyroptosis. To test this possibility, we examined the production and secretion of IL-1β as well as the cleavage of GSDMD, the hallmark of pyroptosis, in THP-1 cells infected with the different MOI ECHO 11 at 24 h.p.i.. THP-1 cells treated with lipopolysaccharides (LPS) plus Nigericin (inducer of NLRP3- and caspase 1-dependent pyroptosis) were used as the positive control. The replication of ECHO 11 and expression of 2B protein in THP-1 cells was examined (S2A and S2B Fig). Our data showed that IL-1β secretion and IL-1β mRNA induction were triggered by ECHO 11 in THP-1 cells in a dose-dependent manner (Fig 2A and 2B). Western blotting analyses uncovered that the cleavages of IL-1β (p17) and GSDMD (N-GSDMD) in the supernatants, and the production of pro-IL-1β in the cell lysates were induced by ECHO 11 infection (Fig 2C). Besides, IL-1β secretion was co-incident with the release of LDH in the supernatants of THP-1 cells (Fig 2D). Moreover, we also found that IL-1β secretion, IL-1β mRNA induction, the cleavages of IL-1β and GSDMD, as well as the release of LDH was induced by ECHO 11 in THP-1 cells in a time-dependent manner (Fig 2E–2H). Therefore, these findings indicate that ECHO 11 infection induces pyroptosis in THP-1 cells.

We further examined the ECHO 11-induced pyroptosis in C57BL/6 mice- differentiated BMDMs, which was also found to support for ECHO 11 replication (S2C and S2D Fig). Similar to the data from THP-1 cells, our data showed that ECHO 11-induced IL-1β secretion and pyroptosis in BMDMs in a dose- and time-dependent manner, which was determined by examining IL-1β secretion, IL-1β mRNA activation, and the accumulation of p17, N-GSDMD, and LDH in cellular supernatants in ECHO 11-infected BMDMs (Fig 2I–2P). Moreover, other inflammatory factors, such as IL-6 and TNF-α, were also found to be upregulated in ECHO 11-infected THP-1 cells and BMDMs (S3A–S3H Fig), confirming that ECHO 11 infection induces inflammation. Together, our findings uncover that ECHO 11 induces inflammation activation and pyroptotic cell death.

### NLRP3-inflammasome is essential for ECHO 11-induced pyroptosis

**Fig 3:**
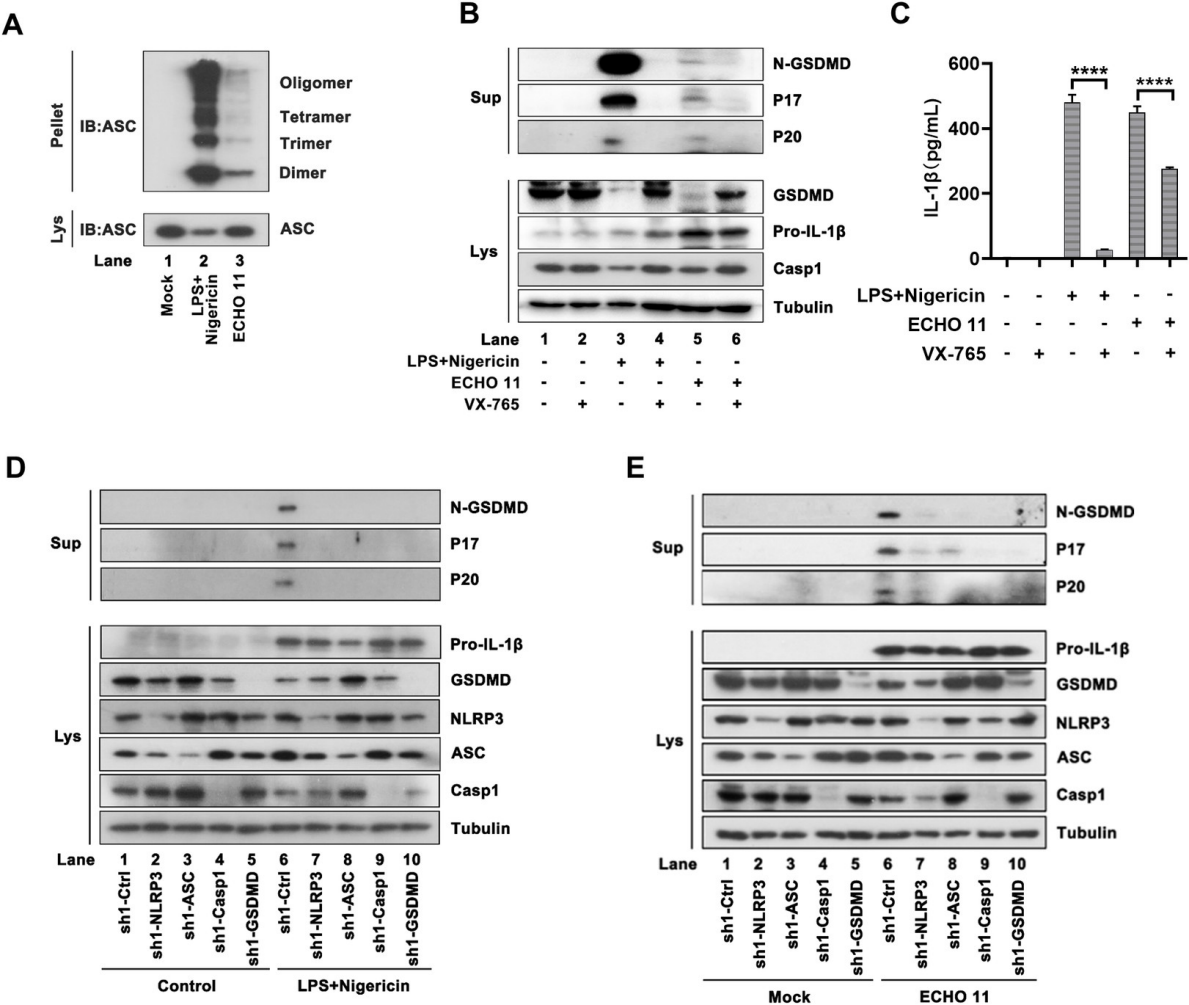
ECHO 11 infection induces pyroptosis through the activation of NLRP3-inflammasomes. (**A**) PMA-differentiated THP-1 macrophages were infected with ECHO 11 at MOI of 0.1, at 24 h.p.i., cells were lysed and the pellets were subjected into cross-link. The ASC oligomerization in the pellets and the total ASC in lysates as the input were examined by Western blotting. Cells treated with LPS/Nigericin were used as the positive control. (**B**) THP-1 cells were infected with ECHO 11 at an MOI of 0.5 for 24 h, and cells treated with CASP-1 inhibitor VX-765 (50 μM) were used as the control. GSDMD-N, P17 and P20 in the supernatants, and GSDMD, pro-IL-1β and CASP-1 in the lysates were examined by Western blotting. (**C**) Mature IL-1β levels in supernatants were determined by ELISA. Data represent means and SD from three repeated experiments. ****, *P* < 0.0001, as measured by two-way ANOVA. (**D-E**) THP-1 cells stably expressing control shRNA (Ctrl) or specific shRNA targeting NLRP3, ASC, CASP-1 and GSDMD were treated with LPS /Nigericin (D) or infected with ECHO 11 at an MOI of 0.5 for 24 h (E). GSDMD-N, P17 and P20 in the supernatants, and GSDMD, pro-IL-1β and CASP-1 in the lysates were examined by Western blotting.

We sought to examine whether ECHO 11-induced pyroptosis was dependent on inflammasome. The oligomerization of the inflammasome adaptor protein, ASC, is an indicator of inflammasome activation [21]. Thus, we examined the effect of ECHO 11 on ASC oligomerization via cross-linking the cell pellet of PMA-differentiated THP-1 macrophages infected with ECHO 11. Oligomerization of ASC was observed in ECHO 11-infected and LPS/Nigericin-treated THP-1 macrophages (Fig 3A), showing the involvement of ASC oligomerization in the formation of ECHO 11-induced inflammasome.

We further examined whether ECHO 11 triggered ASC oligomerization via activating NLRP3 inflammasome. Upon activation, NLRP3 inflammasome promotes auto-cleavage of pro-CASP-1 to mature CASP-1 (p20), which then cleaves the GSDMD and IL-1β. As shown in Fig 3B, the cleavages of CASP-1, IL-1β and GSDMD were activated in THP-1 cells either infected with ECHO 11 or treated with LPS/Nigericin, whereas VX-765 (inhibits the enzymatic activity of CASP-1) treatment abrogated such activations. VX-765 treatment had no effect on ECHO 11 replication (S4 Fig). Inhibition of CASP-1 also significantly suppressed the secretion of IL-1β in the supernatants of ECHO 11-infected THP-1 cells (Fig 3C). Moreover, we constructed THP-1 cell lines stably expressing shRNAs targeting the genes of NLRP3, ASC, CASP-1, which are the key components of NLRP3 inflammasome and GSDMD, respectively. The viral RNA replication of ECHO 11 was not affected by knocking down these genes (S5 Fig). Our data showed that ECHO 11 infection or LPS/Nigericin treatment induced pro-IL-1β maturation, and CASP-1/GSDMD cleavages in THP-1 cells, and these activations were remarkably attenuated by knocking down either one of the NLRP3 inflammasome components and pyroptosis (Figs 3D and 3E and S6). Together, our findings demonstrate that ECHO 11 induces pyroptosis via activating NLRP3 inflammasome.

### ECHO 11-encoded 2B protein activates NLRP3 inflammasome and pyroptosis

We sought to examine the mechanism by which ECHO 11 induced NLRP3 inflammasome-dependent pyroptosis. Thus, the effects of ECHO 11 replication and proteins on the activation of NLRP3 inflammasome and pyroptosis were evaluated. In THP-1 cells, GSDMD and CASP-1 cleavages, IL-1β maturation and secretion, and LDH release were induced by ECHO 11 and LPS/Nigericin, but not by ultraviolet-inactivated (UV-inactivated) or heat-inactivated ECHO 11 that was unable to replicate (S7A–S7J Fig). Moreover, treatment of ECHO 11-infected cells with siRNAs specifically targeting ECHO 11 genomic sequence significantly inhibits the viral RNA replication, coinciding with the substantial reduction in inflammasome activation (S7K–S7O Fig). These findings uncover that ECHO 11 replication is required for NLRP3 inflammasome-dependent pyroptosis.

**Fig 4:**
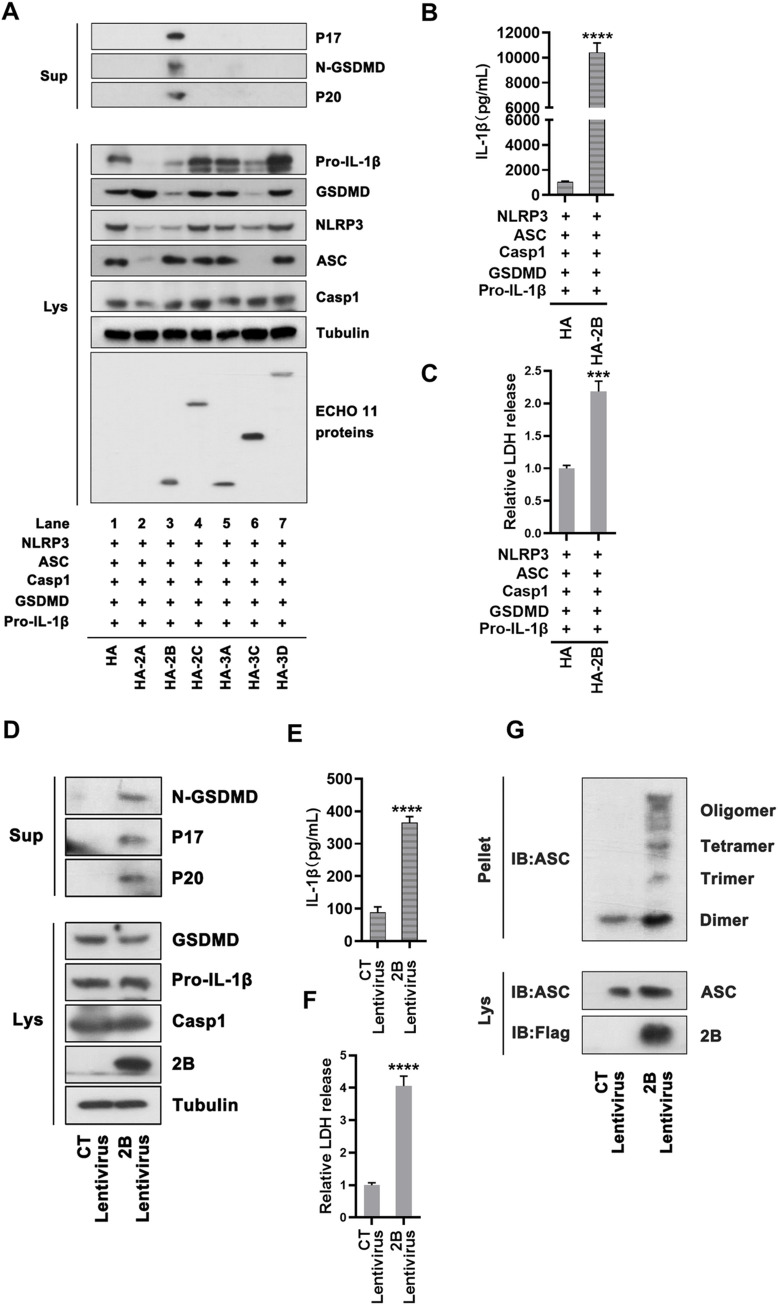
ECHO 11 2B activates NLRP3 inflammasomes and pyroptosis. (**A**) 293T cells were co-transfected with plasmids encoding NLRP3, ASC, CASP-1, Pro-IL-1β and GSDMD together with a vector encoding ECHO 11 non-structure proteins, including 2A, 2B, 2C, 3A, 3C or 3D. GSDMD-N, P20 and P17 in the supernatants, and GSDMD, NLRP3, ASC, CASP-1, viral proteins and pro-IL-1β in the lysates were examined by Western blotting. (**B**) Mature IL-1β levels in the supernatants were determined by ELISA. (**C**) The LDH release in the supernatants were determined by cytotoxicity assay, and the level of LDH in mock cells was defined as 1-fold. (**D**) THP-1 cells were infected with control (CT) or 2B-encoding lentivirus, and then differentiated into macrophages with PMA. GSDMD-N, P17 and P20 in the supernatants and GSDMD, pro-IL-1β, CASP-1 and ECHO 11 2B in the lysates (**E**) Mature IL-1β levels in the supernatants of 2B stably expressing THP-1 cells were determined by ELISA. (**F**) The LDH release in the supernatants were determined by cytotoxicity assay, and the level of LDH in the controlled THP-1 cells was defined as 1-fold. (**G**) The 2B stably expressing THP-1 macrophages were lysed and the pellets were subjected into cross-link. The ASC oligomerization in the pellets and the total ASC in lysates as the input were examined by Western blotting. Data represent means and SD from three repeated experiments. ***, *P* < 0.001; ****, *P* < 0.0001, as measured by unpaired *t* test.

Next, we assessed if any ECHO 11-encoded protein was required for the NLRP3 inflammasome activation. Thus, 293T cells were co-transfected with plasmids encoding GSDMD, NLRP3, ASC, pro-CASP-1 and pro-IL-1β, and then transfected with plasmids encoding each of the six ECHO 11 proteins. Our data showed that expressions of GSDMD-N, P20 and P17 were induced by 2B, but not 2A, 2C, 3A, 3C, or 3D (Fig 4A). The expression of 2A was difficult to detect via Western blotting because enteroviral 2A is a known protease that can cleave a wide-range of host factors and its own expression is restricted from its inhibition effect on host gene expression [22]. We indeed observed that expressions of some components of NLRP3 inflammasome were substantially reduced in the presence of 2A (Fig 4A). Similar results also obtained in cells expressing another viral protease, 3C (Fig 4A). These results are consistent with the previous study that EV71 2A and 3C can inhibit NLRP3 inflammasome via their protease activities [18]. Moreover, the LDH release and IL-1β secretion were effectively activated in the presence of 2B (Fig 4B and 4C). Therefore, these findings indicate that ECHO 11 2B is involved in the activation of NLRP3 inflammasome and pyroptosis.

Furthermore, the role of 2B in regulating the activity of NLRP3 inflammasome was evaluated in THP-1 cells stably expressing 2B via lentivirus transduction. Similar to the data from 293T cells, expression of 2B efficiently activated GSDMD and CASP-1 cleavages, IL-1β maturation/secretion, and LDH release in THP-1 cells macrophages (Fig 4D–4F). Moreover, we also observed that expression of 2B triggered the ASC oligomerization in THP-1 macrophages (Fig 4G). Therefore, our findings uncover that ECHO 11 2B possesses the ability to activate NLRP3 inflammasome and induces pyroptosis.

### ECHO 11 2B interacts with NLRP3 to facilitate inflammasome assembly

**Fig 5:**
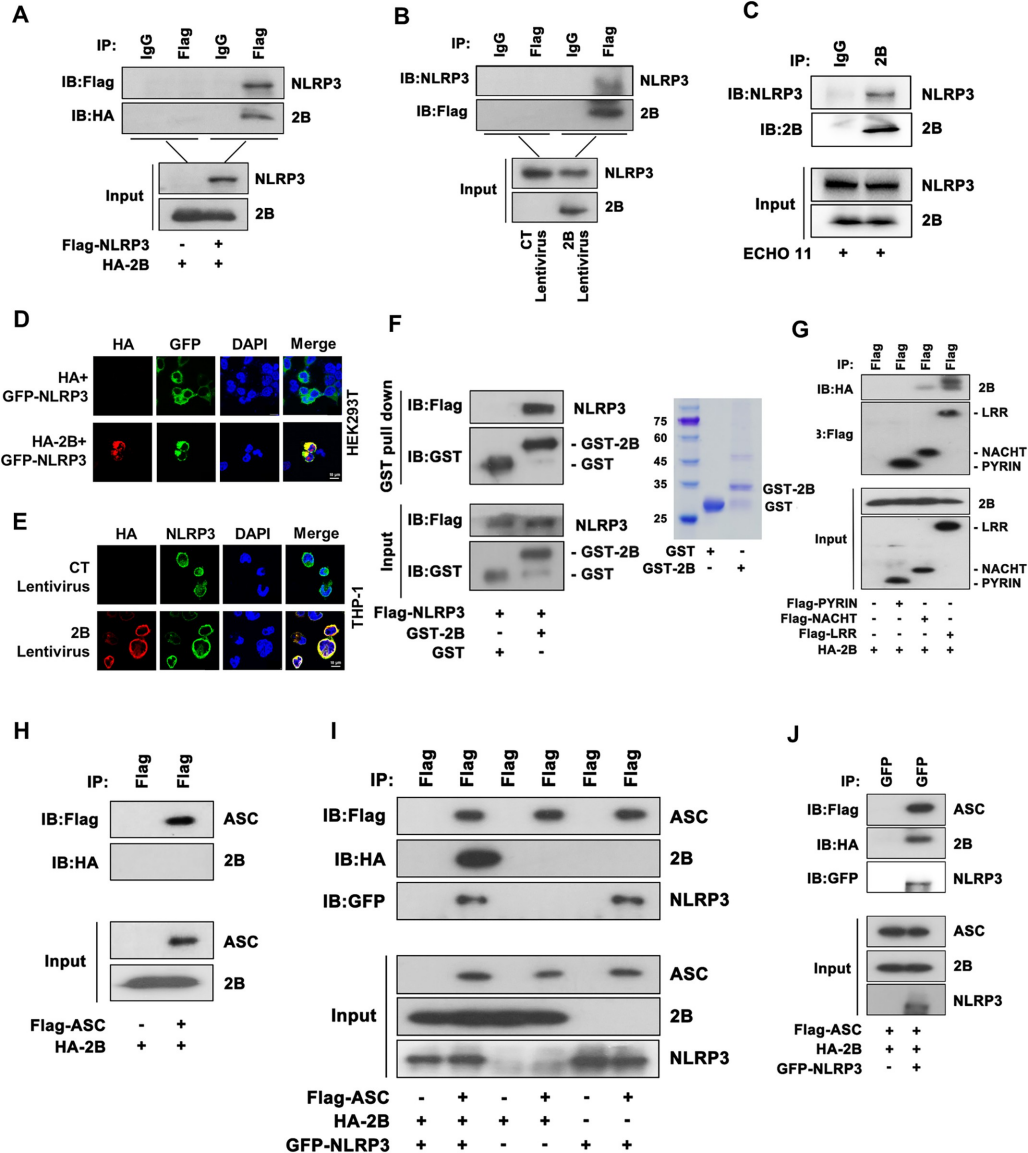
ECHO 11 2B interacts with NLRP3 to facilitate inflammasome assembly. (**A**) 293T cells were co-transfected with plasmids encoding HA-2B and Flag-NLRP3, subjected to Co-IP using anti-Flag antibody or anti-IgG antibody, and analyzed by Western blotting with anti-Flag and anti-HA antibodies. (**B**) THP-1 macrophages stably expressing Flag-2B were subjected to Co-IP using anti-Flag or anti-IgG antibodies, and analyzed by Western blotting with anti-Flag and anti-NLRP3 antibodies. (**C**) THP-1 macrophages were infected with ECHO 11 at an MOI of 0.5 for 24 h, subjected to Co-IP using anti-2B or IgG antibodies, and analyzed by Western blotting with anti-2B and anti-NLRP3 antibodies. (**D-E**) The subcellular localizations of HA-2B (red), GFP-NLRP3 (green), and nucleus marker DAPI (blue) in 293T cells (D) and THP-1 macrophages (E) were analyzed using confocal microscopy. Scale bar, 10 μm. (F) Purified GST, GST-2B, and Flag-NLRP3 were incubated and subject to GST-pulldown followed by immunoblotting with anti-Flag antibody (left). Purified GST, GST-2B were also subjected Coomassie blue staining (right). (**G**) 293T cells were co-transfected with plasmids encoding HA-2B together with Flag-PYRIN, Flag-NACHT, or Flag-LRR. subjected to Co-IP using anti-Flag antibody, and analyzed by Western blotting with anti-Flag and anti-HA antibodies. (**H**) 293T cells were co-transfected with plasmids encoding HA-2B and Flag-ASC, subjected to Co-IP using anti-Flag antibody, and analyzed by Western blotting with anti-Flag and anti-HA antibodies. (**I**) 293T cells were co-transfected with plasmids encoding Flag-ASC together with HA-2B or GFP-NLRP3 or HA-2B plus GFP-NLRP3, subjected to Co-IP using anti-Flag antibody, and analyzed by Western blotting with anti-Flag, anti-HA and anti-GFP antibodies. (**J**) 293T cells were co-transfected with plasmids encoding Flag-ASC, HA-2B and GFP-NLRP3, subjected to Co-IP using anti-GFP antibody, and analyzed by Western blotting with anti-Flag, anti-HA and anti-GFP antibodies.

We further investigated the mechanism by which ECHO 11 2B activated NLRP3 inflammasome. To this end, we examined whether 2B interacted with the key components of NLRP3 inflammasome. Co-immunoprecipitation (Co-IP) assays were conducted with Flag-NLRP3- and HA-2B-expressing 293T cells via using anti-Flag and anti-IgG antibodies, respectively. Our results showed that ECHO 11 2B interacted with NLRP3 in 293T cells (Fig 5A). The association of 2B and NLRP3 was further confirmed in THP-1 macrophages stably expressing 2B (Fig 5B). Moreover, endogenous Co-IP experiment using anti-2B antibody also verified that ECHO 11 2B interacted with NLRP3 in THP-1 macrophages in the context of ECHO 11 infection (Fig 5C). The interaction of 2B with NLRP3 was further examined via confocal microscopy in 293T and HeLa cells, and THP-1 macrophages expressing HA-2B and GFP-NLRP3 (Figs 5D and 5E and S8A). NLRP3 and 2B are both membrane-associated proteins. Thus, to exclude the possibility that the NLRP3-2B interaction is mediated through non-specific membrane interaction, we applied another membrane-bounding NLR, NLRP6, as a negative control in the Co-IP assay. Our results showed that ECHO 11 2B failed to interact with NLRP6 (S8B Fig), indicating that 2B interacts with NLRP3 through the protein-protein interaction but not membrane association. In addition, we also determined the 2B-NLRP3 interaction via GST pull-down assay, in which the purified GST-fusion 2B was incubated with Flag-NLRP3 purified from the lysates of 293T cells, indicating that the direct interaction between ECHO 11 2B and NLRP3 (Fig 5F).

NLRP3 consists of an N-terminal PYRIN (PYD) domain, a central nucleotide-binding and oligomerization (NACHT) domain and a C-terminal leucine-rich repeat (LRR) domain. We then examined which domain was required for the NLRP3-2B interaction via Co-IP assays using 293T cells expressing HA-2B together with Flag-PYRIN, Flag-NACHT or Flag-LRR, respectively. Our results showed that 2B was interacted with NACHT and LRR domains, but not PYD domain (Fig 5G). Therefore, ECHO 11 2B binds to NLRP3 through interacting with the NACHT and LRR domains.

Upon activation, the oligomerized NLRP3 interacts with ASC via its PYD domain, which in turn results in the recruitment of pro-CASP-1 and auto-cleavage to form activated CASP-1. After identifying that ECHO 11 2B interacts with NLRP3 and activates ASC oligomerization, we sought to examine whether ECHO 11 2B also interacted with ASC. Thus, 293T cells expressing HA-2B and Flag-ASC were subjected to Co-IP assay and the results showed that ECHO 11 2B failed to interact with ASC (Fig 5H). Moreover, Co-IP assays with 293T cells expressing HA-2B together with Flag-ASC and/or GFP-NLRP3 found that ECHO 11 2B was associated with ASC only in the presence of NLRP3 (Fig 5I and 5J), indicating a 2B-NLRP3-ASC structure formation. Furthermore, the observation that the NLRP3-ASC interaction was not enhanced by 2B (Fig 5I) suggest that the mechanism of ECHO 11 2B to activate NLRP3 inflammasome is via inducing NLRP3’s conformal change but not stimulating the NLRP3-ASC interaction.

Previous study has shown that the increase in Ca^2+^ flux from intracellular storages to the cytosol induced by encephalomyocarditis virus (EMCV) 2B activates NLRP3 inflammasome [23]. To determine if ECHO 11 2B can also activate NLRP3 inflammasome via this similar mechanism, we examined the intracellular Ca^2+^ flux in 2B-expressing THP-1 macrophages via flow cytometry with the calcium-sensitive dye Fluo-4 acetoxymethyl ester (Fluo-4/AM). The stable EMCV 2B-expressing THP-1 macrophages were also constructed as the positive control. The expression of ECHO 11 2B indeed stimulated the intracellular Ca^2+^ flux (S9A Fig), confirming the conserved function of ECHO 11 2B as a enteroviral viroporin. Moreover, we used BAPTA-AM, a well-known membrane permeable Ca^2+^ chelator, to inhibit the accumulation of the intracellular Ca^2+^, and showed that BAPTA-AM efficiently reduced ECHO 11 and EMCV 2Bs-induced Ca^2+^ flux in a dose-dependent manner (S9A and S9B Fig). The inflammasome activation was expectedly inhibited by BAPTA-AM in EMCV 2B-expressing THP-1 macrophages (S9C-S9E Fig). In contrast, BAPTA-AM treatment had no effect on inflammasome in ECHO 11 2B-expressing THP-1 macrophages (S9C–S9E Fig), showing that the intracellular Ca^2+^ flux is irrespective of ECHO 11 2B-activated inflammasome. Our findings indicate that the activity of ECHO 11 2B to stimulate Ca^2+^ flux is not relevant to its inflammation activation.

Together, our findings demonstrate that ECHO 11 2B promotes the assembly of NLRP3 inflammasome via interacting with NLRP3. We speculate that the interactions of ECHO 11 2B with the NACHT and LRR domains of NLRP3 may result in the conformal change of NLRP3 PYD domain, which then enables to recruit ASC and trigger the ASC oligomerization, thereby activating NLRP3 inflammasome.

### I65 and V67 are the key sites for ECHO 11 2B activating NLRP3 inflammasome

**Fig 6:**
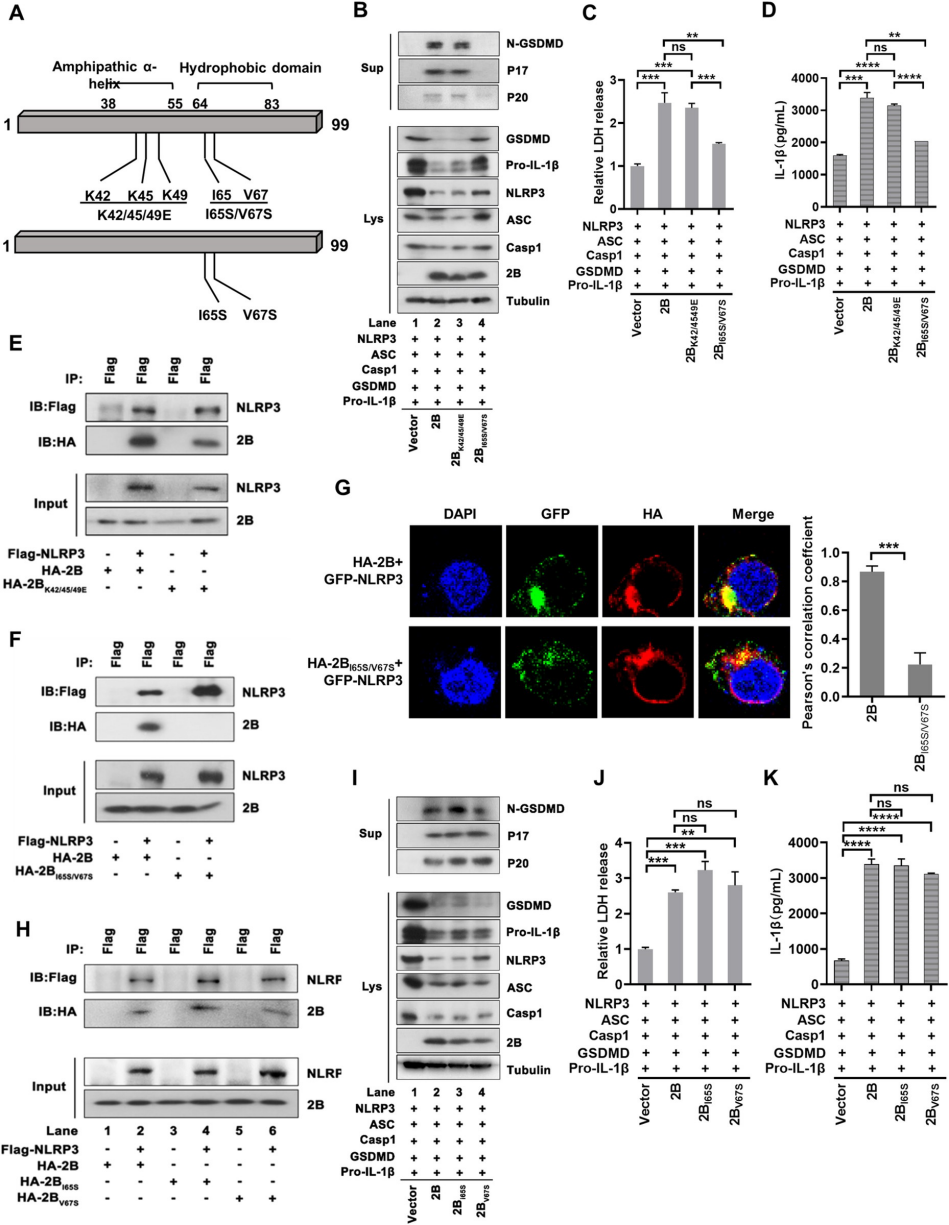
I65 and V67 are the key sites for ECHO 11 2B activating NLRP3 inflammasome. (**A**) Schematic representation of two ECHO 11 2B mutants, K42/45/49E and I65S/V67S. (**B-D**) 293T cells were co-transfected with plasmids encoding NLRP3, ASC, CASP-1, GSDMD, pro-IL-1β, together with the empty vector, 2B_WT_, 2B_K42/45/49E_ or 2B_I65S/V67S_. GSDMD-N, P20 and P17 in the supernatants, and GSDMD, NLRP3, ASC, CASP-1, pro-IL-1β and 2B in the lysates were examined by Western blotting. (**C**) The LDH release in the supernatants were determined by cytotoxicity assay, and the level of LDH in cells transfected with the empty vector was defined as 1-fold. (**D**) Mature IL-1β levels in supernatants were determined by ELISA. Data represent means and SD from three repeated experiments. **, *P* < 0.01; ***, *P* < 0.001; ****, *P* < 0.0001; ns, not significant, as measured by one-way ANOVA. (**E**) 293T cells were co-transfected with plasmids encoding Flag-NLRP3 together with HA-2B_WT_, HA-2B_K42/45/49E_, subjected to Co-IP using anti-Flag antibody, and analyzed by Western blotting with anti-Flag and anti-HA antibodies. (**F**) 293T cells were co-transfected with plasmids encoding Flag-NLRP3 together with HA-2B_WT_, or HA-2B_I65S/V67S_, subjected to Co-IP using anti-Flag antibody, and analyzed by Western blotting with anti-Flag and anti-HA antibodies. (**G**) 293T cells were co-transfected with plasmids encoding GFP-NLRP3 together with HA-2B, or HA-2B_I65S/V67S_. The co-localization of 2B or 2B_I65S/V67S_ with NLRP3 was analyzed by confocal microscopy, and quantified as Pearson’s correlation coefficient with Image J software. Scale bar, 5 μm. ***, *P* < 0.001, as measured by unpaired *t* test. (**H**) 293T cells were co-transfected with plasmids encoding Flag-NLRP3 together with HA-2B_WT_, HA-2B_I65S_ or HA-2B_V67S_, subjected to Co-IP using anti-Flag antibody, and analyzed by Western blotting with anti-Flag and anti-HA antibodies. (**I**) 293T cells were co-transfected with plasmids encoding NLRP3, ASC, CASP-1, GSDMD, pro-IL-1β, together with the empty vector, 2B_WT_, 2B_I65S_ or 2B_V67S_. GSDMD-N, P20 and P17 in the supernatants, and GSDMD, NLRP3, ASC, CASP-1, pro-IL-1β and 2B in the lysates were examined by Western blotting. (**J**) The LDH release in the supernatants were determined by cytotoxicity assay, and the level of LDH in cells transfected with the empty vector was defined as 1-fold. (**K**) Mature IL-1β levels in supernatants were determined by ELISA. Data represent means and SD from three repeated experiments. **, *P* < 0.01; ***, *P* < 0.001; ****, *P* < 0.0001; ns, not significant, as measured by one-way ANOVA.

We sought to determine the key amino acid residues responsible for ECHO 11 2B activating NLRP3 inflammasome. The viral 2B is conserved among EV-B and previous genetic analysis has shown that CVB3 2B contains a cationic amphipathic α-helix and a hydrophobic region [24], which is also observed in ECHO 11 2B (Fig 6A). Moreover, multiple mutations into the amphipathic α-helix (aa 37–54) or hydrophobic region (aa 63–80) of CVB3 2B, i.e., K42/45/49 or I65/V67, have been reported to be related to the CVB3 2B’s activity [24]. Thus, we introduced these multiple mutations into ECHO 11 2B (Fig 6A). Plasmids that encoded wild-type (WT) and mutant ECHO 11 2B proteins were co-transfected with GSDMD, NLRP3, ASC, pro-Casp1, and pro-IL-1β into 293T cells and assayed for examining the downstream events of the NLRP3 inflammasome activation and pyroptosis. Our data showed that I65S/V67S, but not K42/45/49/E mutation, remarkably attenuated the NLRP3 inflammasome activation induced by 2B (Fig 6B–6D). We further examined the effect of I65S/V67S mutations on the 2B-NLRP3 interaction. Co-IP assays showed that, unlike with 2B_WT_ and 2B_K42/45/49/E_, 2B_I65S/V67S_ was unable to interact with NLRP3 (Fig 6E and 6F). Immunofluorescence assay also confirmed that the interaction of 2B_I65S/V67S_ with NLRP3 was substantially inhibited when being compared with that of 2B_WT_ (Fig 6G). Moreover, mutation of either site alone at I65 and V67 (i.e., I65S and V67S) had no effect on the 2B-NLRP3 interaction and NLRP3 inflammasome activation (Fig 6H–6K). Therefore, our findings indicate that the interaction of ECHO 11 2B and NLRP3 is required for the NLRP3 inflammasome activation, and both I65 and V67 are the key residues for 2B interacting with NLRP3.

### ECHO 11 2B induces NLRP3 inflammasome activation in mice

**Fig 7:**
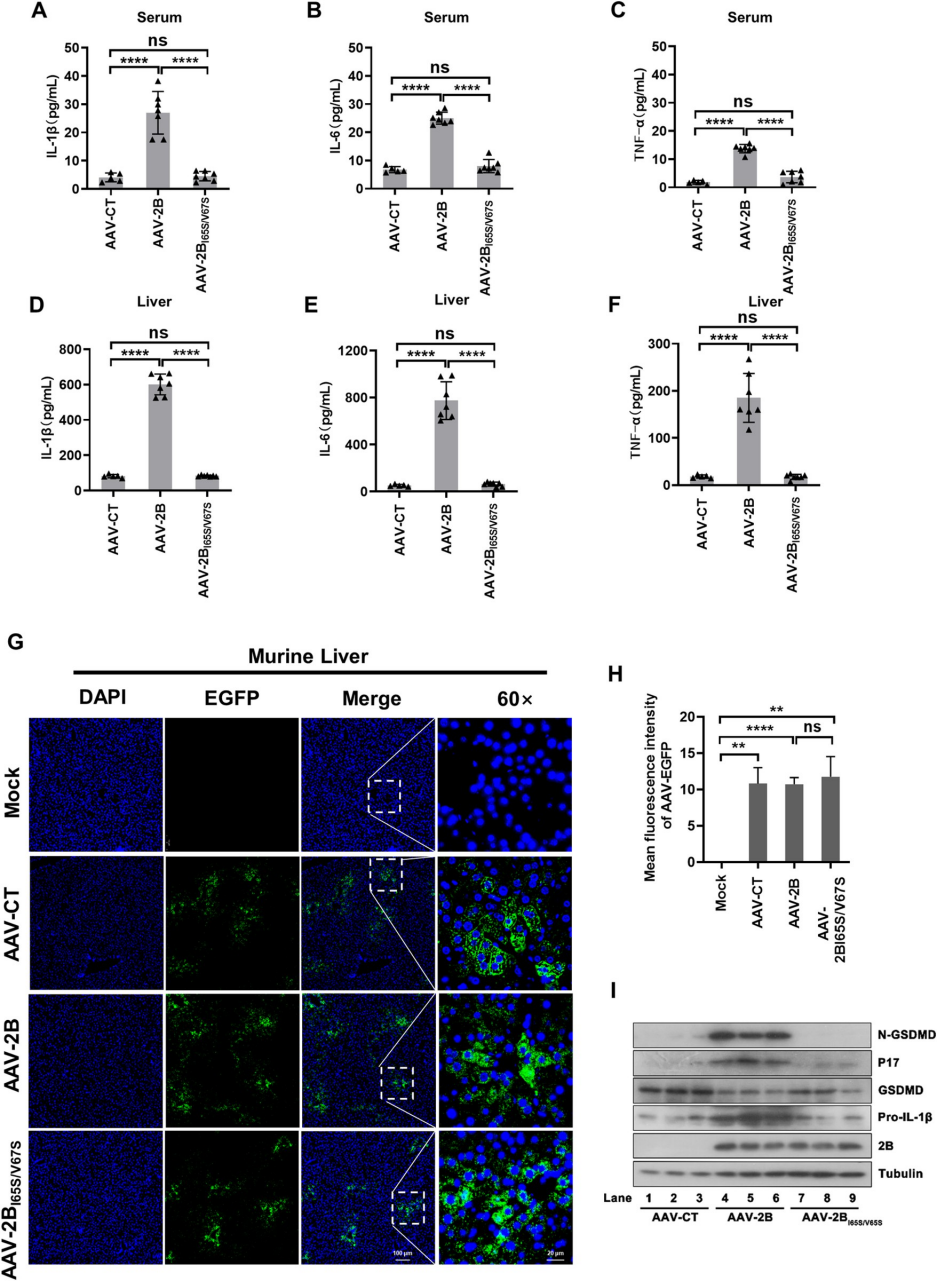
ECHO 11 2B induces NLRP3 inflammasome activation in murine liver. (**A-C**) C57BL/6 mice were tail vein-injected 4 × 10^11^ VG of AAV2/9-EGFP-2B-Flag (AAV-2B) (*n* = 7), AAV2/9-EGFP-2B_I65S/V67S_-Flag (AAV-2B2B_I65S/V67S_) (*n* = 7) or AAV2/9-EGFP (AAV-CT) (*n* = 5) for 3 weeks. (**A-F**) Serum and livers was collected at 3 weeks for each group from the orbit. IL-1β, IL-6, and TNF-αin the serum and livers was measured by ELISA. Points represent the value of each sample. (**G**) Immunofluorescence analysis of EGFP and EGFP-fused 2B (green), and DAPI (blue) in the liver tissue sections. Scale bar is 100 μm (10×) or 20 μm (60×). (**H**) The fluorescence intensity of AAV-EGFP in each group was quantified using Image J software. Data represent means and SD, **, *P* < 0.01; ****, *P* < 0.0001; ns, not significant, as measured by one-way ANOVA. (**I**) GSDMD-N, P17, GSDMD, and pro-IL-1β in the livers of different groups were analyzed by Western blotting. The *in vivo* experiment was repeated three times and obtained similar results.

We further assessed the physiological relevance of ECHO 11 2B-induced NLPR3 inflammasome activation and pyroptosis *in vivo*. Since no animal model of ECHO 11 infection is available, we administered adult C57BL/6 mice with adeno-associated virus (AAV) encoding EGFP-fused 2B (AAV-2B), 2B mutant (AAV-2B_I65S/N67S_) or EGFP alone (AAV-CT) as a negative control via vein injection, respectively. Moreover, considering that fulminant hepatic failure was the dominant clinical feature of fatal disease caused by ECHO 11 and liver cells supported for ECHO 11 infection, we focused on the effects of 2B expression on murine liver tissues and serum cytokines. The concentrations of IL-1β, IL-6 and TNF-α in the serum and liver tissue were remarkably stimulated in AAV-2B-challenged mice, but not in AAV-2B_I65S/N67S_-challenged or control mice (Fig 7A–7F). In agreement, expression of WT 2B efficiently activated IL-1β maturation and GSDMD cleavage in murine liver, whereas 2B_I65S/N67S_ had no effect on inflammasome activation (Fig 7G–7I), consistent with the data obtained using cell lines. These results confirm that ECHO 11 2B activates NLRP3 inflammasome in murine liver, and I65 and V67 are the key residues for 2B to action.

In addition, AAV-challenged mice were treated with Ac-YVAD-cmk (CASP-1 inhibitor) or MCC950 (NLRP3 inhibitor) via intraperitoneal injection. Treatment with Ac-YVAD-cmk or MCC950 substantially inhibited NLRP3 inflammasome activation (S10A–S10D Fig). Similar results were obtained in the spleen that was determined by immunohistochemistry analysis (IHC) of N-GSDMD (S11 Fig). Thus, our findings indicate that ECHO 11 2B can effectively induce the activation of NLRP3 inflammasome and pyroptosis in mice.

### The 2Bs of multiple EVs induce pyroptosis by interacting with NLRP3

After identifying that ECHO 11 2B can activate NLRP3 inflammasome and pyroptosis, it is intriguing to examine whether 2Bs from other EVs possess a similar activity. Thus, we examined the NLRP3 inflammasome activation and pyroptosis in 293T cells expressing 2Bs from EV71, CVA16 and CVB3, respectively. Our data showed that all 2Bs can trigger GSDMD cleavage, CASP-1 maturation, IL-1β maturation/secretion, and LDH release in 293T cells (S12A–S12C Fig). Moreover, Co-IP assays showed that 2Bs from EV71, CVA16 and CVB3 can also interact with NLRP3 as ECHO 11 2B did (S12D Fig). Therefore, our findings uncover that multiple enteroviral 2Bs can induce pyroptosis by interacting with NLRP3.

## Discussion

The inflammasome pathway and its downstream cytokines play critical roles in EV-induced inflammation, whereas the mechanism by which EVs trigger inflammation is less understood. Here we showed that ECHO 11 can induce inflammatory responses via triggering NLRP3 inflammasome activation and pyroptosis in THP-1 cells and mouse BMDMs. Expression of ECHO 11-encoded 2B was sufficient to activate NLRP3 inflammasome in cells and *in vivo*, and the interaction between 2B and NLRP3 was required for inflammasome complex assembly. Thus, our work provides evidence that ECHO 11 can induce inflammatory responses through activating NLRP3 inflammasome by 2B.

NLRP3 consists of an N-terminal PYD domain, a central NACHT domain and a C-terminal LRR domain, of which the NACHT domain regulates oligomerization and serves as a scaffold for inflammasome assembly [25]. The PYD domain mediates signal transduction via interacting with other proteins containing PYD domains, and LRR domain serves as a ligand sensor of external stimuli. Upon activation, NLRP3 oligomerizes via homotypic interactions between NACHT domains, thereby leading to the conformal change of its PYD domain to interact with the PYD domain of ASC, which recruits ASC to NLRP3 and results in the formation of ASC prion-like oligomerizes. This, in turn, enables the recruitment of pro-CASP-1 to inflammasome and triggers its auto-cleavage to form activated CASP-1 [10,26]. Our findings showed that the 2B-NLRP3 interaction triggered the ASC oligomerization and NLRP3 inflammasome activation, indicating that 2B enables the assembly of the 2B–NLRP3–ASC complex. Moreover, ECHO 11 2B was associated with ASC only in the presence of NLRP3, and the interaction of NLRP3 and ASC was not enhanced by 2B, suggesting that the mechanism of 2B to activate inflammasome is via inducing NLRP3’s conformal change but not stimulating the NLRP3-ASC interaction. Considering that ECHO 11 2B can directly interact with the NACHT and LRR domains of NLRP3, but not ASC, it is postulated that the interactions of ECHO 11 2B with the NACHT and LRR domains of NLRP3 may directly result in the conformal change of NLRP3 and allow PYD domain to recruit ASC, leading to the ASC oligomerization and activating NLRP3 inflammasome.

Enteroviral 2B is a viroporin that forms pores on the cellular membrane to mediate ion efflux. Ito et al. demonstrated that encephalomyocarditis virus (EMCV) 2B is sufficient to activate NLRP3 inflammasome by enhancing cytosolic Ca^2+^ [23]. Moreover, the 2B-enhanced permeability of cellular membrane and inflammasome activation has also been observed in human rhinovirus (HRV) [27]. However, Wang et al. found that expression of EV71 2B cannot trigger NLRP3 inflammasome activation [17]. Here we uncover that ECHO 11 2B activates NLRP3 inflammasome via directly interacting with NLRP3 to facilitate the inflammasome complex assembly. Moreover, although ECHO 11 2B indeed stimulated the intracellular Ca^2+^ flux, the intracellular level of Ca^2+^ was irrelevant to 2B-induced inflammasome, as inhibition of the intracellular Ca^2+^ accumulation by Ca^2+^ chelator had no effect on inflammasome activation by 2B. These results indicate that ECHO 11 2B-activated inflammasome is irrespective of its activity to stimulate the intracellular Ca^2+^ flux. Thus, our findings provide a novel insight into the mechanism of the enteroviral 2B-induced inflammation. We also showed that 2Bs of EV71, CVA16 and CVB3 can interact with NLRP3 and induce pyroptosis, consistent with the previous observations that poliovirus and EV71 2B induce the cellular redistribution of NLRP3 [28]. These findings suggest that enteroviral 2B may trigger NLRP3 inflammasome via different mechanisms, and the mechanism of action of 2B-induced inflammation from distinct EVs may be varied.

In addition to 2B, other components of EV have also been found to mediate the activation of NLRP3 or other inflammasomes. EV71 3D can stimulate the activation of NLRP3 inflammasome and induce the secretion of IL-1β [17]. Moreover, absent in melanoma 2 (AIM2) inflammasome can be triggered by cytosolic EV71 RNA in neuronal cells [29]. These results indicate that different inflammasomes can be activated by various components of one pathogen. Future studies should examine whether ECHO 11 RNA activates other inflammasomes. Besides, enteroviral 2A and 3C have been identified to inhibit the activation of NLRP3 inflammasome via cleaving the NLRP3 and/or GSDMD [22,30]. We observed that expressions of some components of NLRP3 inflammasome were substantially reduced by ECHO 11 2A and 3C, suggesting that 2A and 2C may inhibit NLRP3 inflammasome activation. Notably, under the context of viral infection, ECHO 11 effectively induces NLRP3 inflammasome activation in the presence of 2A and 3C. These findings suggest that 2B may confer a powerful inflammasome activation, which would be negatively controlled but not completely inhibited by 2A and 3C, during ECHO 11 infection. Future studies should investigate this possibility. Moreover, the activation of NLRP3 inflammasome is tightly regulated by distinct EV-encoded proteins via different mechanisms, highlighting the importance of NLRP3 inflammasome during EVs infection.

The activation of NLRP3 inflammasome is important for host defense against viruses [9,31], while excessive inflammation and pyroptosis can be detrimental, which contributes to severe viral immunopathology [32–34]. Acute liver failure was a usually identified symptom in severe ECHO 11 infections in neonates, and inflammation is a major cause of the pathogenesis of acute liver damage in many cases [4–8]. We showed that ECHO 11 can efficiently infect human liver cells and macrophages, and induce potent inflammation in these cells. Moreover, ECHO 11 2B induced NLRP3 inflammasome activation in hepatic cells, intrahepatic macrophages and murine livers, which is consistent with the clinical observations that ECHO 11 viral RNA can be detected in the liver samples of newborn patients who suffered necrotizing hepatitis associated with excessive inflammation. Thus, we speculate that 2B is an important contributing factor for inducing exacerbated inflammation during ECHO 11 infection. The inflammatory responses would worsen hepatic damage and aggravate liver inflammation, leading to severe disease and even fatal outcomes. Moreover, our findings showed that MCC950 treatment remarkably attenuated ECHO 11-induced inflammation, suggesting that an effective NLRP3 inhibitor may improve patient outcomes. Future studies should elucidate this promising immunotherapeutic strategy for the treatment of enteroviral diseases.

In conclusion, this work provides the first demonstration that ECHO 11 can induce inflammatory responses via activating NLRP3 inflammasome and pyroptosis. It also uncovers the mechanism by which ECHO 11-encoded 2B interacts with NLRP3 to induce NLRP3 inflammasome complex assembly. These findings extend our knowledge about ECHO 11 and its encoded 2B, and shed light on the pathogenicity of ECHO 11.

## Materials and methods

### Ethics statement

All work performed in this study was approved by the Guangzhou Women and Children’s Medical Center Ethics Committee (NO. 274A01). Written informed consent was waived by the Ethics Commission of the Guangzhou Women and Children’s Medical Center for emerging infectious diseases.

### Human subjects

Patients A and B are newborn twins, and appeared normal and breastfed well for the first 6 days after birth. At the age of postnatal day 7, both patients presented recurrent vomiting, decreased urine output, low blood pressure, coagulation dysfunction and shock. Then they were transferred to the pediatric intensive care unit in our medical center at postnatal day 8 and underwent jugular catheterization for continuous renal replacement therapy. Fresh frozen plasma, coagulation factor, red blood cell (RBC), platelet, prothrombin, fibrinogen and immunoglobulin were administered. However, the clinical condition of the patients progressively deteriorated and both patients appeared the opposite levels between serum bilirubin and ALT at postnatal day 15. Both patients developed to extensive skin ecchymosis and represented status of no urine output. The liver of patient A was 2.5 cm below the right costal margin. Cranial B-ultrasound showed that patient A presented right parietal lobe hemorrhage, subarachnoid hemorrhage, brain edema, bilateral ependymal cysts and cerebral longitudinal fissure widened. Patient B presented thalamus hemorrhage, bilateral ependymal cysts, brain edema, and multiple small necrotic foci in brain parenchyma. Patient A died of hemorrhage at postnatal day 20 and Patient B died of hemorrhage at postnatal day 26.

ECHO 11 viral RNA was detected via qRT-PCR in throat and nasal swab samples at postnatal days 8 and 18 for patient A, and at postnatal days 8, 15 and 18 for patient B. ECHO 11 viral RNA was detected via qRT-PCR as previously reported [35]. We excluded the potential infections by other pathogens, including Enterovirus A, adenovirus, herpes simple virus, Epstein-Barr virus, cytomegalovirus, hepatitis B virus, hepatitis C virus, human immunodeficiency virus, respiratory syncytial virus, and Yersinia pseudotuberculosis via examining the serum and swab samples from the patients. Liver tissues of both patients were collected via postmortem needle puncture, and ECHO 11 viral RNA was detected via using the ECHO 11 Nucleic Acid Detection Kit (JC20118) purchased from bioPerfectus Technologies according to the manufacture’s instructions.

### Cell lines and cultures

HEK293T, RD, Vero, THP-1, Huh7, HepG2 and HeLa cell lines were commercially obtained from ATCC. HEK293T, Vero, RD, and HeLa cell lines were maintained in Dulbecco’s modified Eagle’s medium (DMEM) (Gibco) supplemented with 10% fetal bovine serum (FBS) (Gibco), 100 U/mL penicillin and 100 μg/mL streptomycin (Hyclone). THP-1 cells were cultured in RPMI 1640 medium (Gibco) supplemented with 10% FBS, 100 U/ml penicillin, and 100 μg/mL streptomycin sulfate. Cells were maintained in an incubator at 37°C with 5% CO_2_. THP-1 cells were differentiated into macrophages by treatment with 40 ng/mL of PMA for 24 h at 37°C as previously reported [36–38]. Mouse BMDMs were differentiated from fresh bone marrow of C57BL/6 mice. The bone marrow cells were incubated in six-well plates for 7 days with 20 ng/mL M-CSF and 10% FBS in RPMI 1640 medium. The culture medium was replaced every 2 days [39,40].

### Reagents and antibodies

Lipopolysaccharide (LPS) (L9641), Phorbol-12-myristate-13-acetate (PMA) (P1585), Ac-YVAD-cmk (SML0429) and Disuccinimidyl suberate (DSS) (S1885) were purchased from Sigma-Aldrich. Recombinant murine M-CSF (315–02) was purchased from PeproTech. Puromycin (abs9143) was purchased from Absin. RPMI-1640 and DMEM were obtained from Gibco. Nigericin (tlrl-nig) was obtained from InvivoGene. VX-765 (S2228) and MCC950 (S7809) was purchased from Selleck. Antibody against Flag (M185-7) (1:2000) and monoclonal mouse anti-HA (M180-7) (1:5000) were purchased from MBL. Monoclonal rabbit anti-NLRP3 (D2P5E) (1:1000), monoclonal rabbit anti-GSDMD (E9S1X) (1:1000), monoclonal rabbit anti-cleaved GSDMD (Asp276) (E3E3P) (1:1000), monoclonal rabbit anti-Caspase-1 (E2Z1C) (1:1000), monoclonal rabbit anti-Cleaved Caspase-1 (Asp296) (E2G2I) (1:1000) were purchased from Cell Signaling Technology. Monoclonal mouse anti-ASC (sc-271054) (1:1000) were purchased from Santa Cruz Biotechnology. Polyclonal rabbit anti-IL-1β (ab9722) (1:1000) were purchased from Abcam. Monoclonal anti-HRP-conjugated α-Tubulin (HRP-66031) (1:5000) was purchased from Proteintech. Human IL-1β ELISA Kit (DLB50) and Mouse IL-1β ELISA Kit (MLB00C) were purchased from R&D Systems. LDH Cytotoxicity Assay Kit (C20300) was purchased from Invitrogen.

### Viruses

The ECHO 11 strain GWCMC01/GZ/CHN/2019 (Genebank No. MN817130) was propagated in RD cells and used in this study. Viral titer was measured using the 50% tissue culture infectious dose (TCID_50_) in Vero cells, and the PFU was calculated following that 0.7 PFU is equivalent to 1 TCID_50_ [41–43]. For the preparation of UV-inactivated ECHO 11, the virus was dispersed in a tissue culture dish, and a compact UV lamp was placed directly above the dish for 30 min. For the preparation of heat-inactivated ECHO 11, the virus was treated for 1 h at 60°C. To inhibit ECHO 11 replication, two siRNAs were transfected into cells 24 h before viral infection. siRNA1: GCTAGTCAAGATCATCTCA; siRNA2: CAGCCATGCTAGCCCTTAT.

### RNA sequencing and analysis

The extracted RNA was digested and fragmented, end repaired, and followed by adaptor ligation and PCR amplification to construct the library. The Agilent 2100 Bioanalyzer quality control library fragment size was about 300 bp, and the Qubit dsDNA HS Assay Kit (Thermo Fisher Scientific Inc.) was used. The concentration of DNA libraries is controlled, and the constructed libraries are pooled in equal masses according to the detected concentration. The pooled libraries are looped to form a single-stranded loop structure. After rolling circle replication (RCA), DNB nanospheres are generated. The prepared DNB nanospheres are loaded onto the sequencing chip, and BGISEQ-50/MGISEQ-2000 is used for sequencing [44].

A total of 4,918,963 sequencing reads with single-end 100 bp length were aligned to a full set of 43 complete echovirus references obtain by NCBI Nucleotide database (updated data: 2019/05/15) in BWA-MEN model (BWA: http://bio-bwa.sourceforge.net/) with default parameter. References present with the highest coverage (EF634316.1) were then choose and supplied as trusted contigs to SPAdes (V3.1.1) for assembling. Assembling contigs were then annotated by BLASTN (V2.7.1+) and the best hit result was used. All assembling contigs was re-order to 5’-3’ forward and sorted by reference starting to point [45].

### Plasmid construction

The plasmids encoding NLRP3 and NLRP6 were constructed with pRK-flag and pEGFP-C1 vectors, respectively. The plasmid encoding ASC was constructed with pRK-flag vector. The plasmid encoding IL-1β was constructed with pCAggs vector. The plasmids expressing GSDMD using pCMV-flag vector (HG25207-NF) and CASP-1 using pCMV-HA vector (HG11148-NY) were purchased from Sino Biological. To construct plasmids expressing ECHO 11 proteins 2A, 2B, 2C, 3A, 3C, and 3D, corresponding fragments of ECHO 11 cDNA were cloned into pCAggs-HA vector. The PYD, NACHT, and LRR domain of NLRP3 was cloned into pRK-flag vector, respectively.

### Lentivirus-mediated gene stable expression and knockdown

Knockdown of NLRP3, ASC, CASP-1, and GSDMD was performed via lentiviral transduction in THP-1 cells. The targeting sequences of shRNAs for human NLRP3, ASC, and CASP-1 were as follows: sh1NLRP3: 5’-CCGTAAGAAGTACAGAAAGTA-3’; sh1ASC: 5’-GCCCACCAACCCAAGCAAGAT-3’; sh1CASP-1: 5’-CACACG TCTTGCTCTCATTAT-3’; sh1GSDMD: 5’-GTGTGTCAACCTGTCTATCAA-3’. sh2NLRP3: 5’-CAGGTTTGACTATCTGTTCT-3’; sh2ASC: 5’- GATGCGGAAGCTCTTCAGTTTCA-3’; sh2CASP-1: 5’- GTGAAGAGATCCTTCTGTA-3’; sh2GSDMD: 5’- CCTTCTCTTCCCGGATAAGAA-3’; The pLKO.1 vector encoding non-specific shRNA as a negative control or a specific shRNA targeting one of the NLRP3 components as noted above was transfected into 293T cells together with CG8.91 and PMD2.G. For stably expressing ECHO 11 or EMCV 2B in THP-1 cells, 2B ORF was cloned into phage-3xFlag vector, named phage-2B-3xFlag, and then transfected into 293T cells with the packaging vectors pSPAX2 and pMD2G. Culture supernatants were harvested after transfection at 48 h.p.i.. THP-1 cells were then infected with the supernatants containing lentiviral particles. After 48 h, cells were selected by 2 μg/ml puromycin for 2 days, and the knocking down effects were detected via Western blotting.

### Western blot analysis

Cells treated as indicated were lysed with buffer (1% Triton X-100, 0.5% sodium deoxycholate, 0.1% SDS, 50 mM Tris, pH 7.5, 150 mM NaCl, 5 mM EDTA, and 10% glycerol). Cell supernatants were incubated with Trichloroacetic acid (TCA) overnight at 4°C and centrifuged at 12000 g for 10 min at 4°C. The pellets were washed, mixed with SDS loading buffer and boiled for 10 min for analysis. Cell lysates and supernatants were subjected to 10%-12% SDS-polyacrylamide gel electrophoresis (PAGE) and then transferred to polyvinylidene difluoride (PVDF) membranes (Millipore). Then, the membranes were blocked with 5% nonfat milk in TBST buffer (50 mM Tris/HCl, pH 7.4, 150 mM NaCl and 0.1% Tween-20) for 1–2 h, followed by incubation in primary antibody diluted in 5% nonfat milk-TBST for 12 h at 4°C. Afterward, the membranes were incubated with the appropriate horseradish peroxidase (HRP)-conjugated secondary antibody diluted in 5% nonfat milk-TBST for 1 h. The expression of antigen was visualized using an enhanced chemiluminescence (ECL) kit (Millipore).

### Co-immunoprecipitation assays

Cell lysates were prepared by using lysis buffer (1% Triton X-100, 0.5% sodium deoxycholate, 50 mM Tris, pH 7.5, 150 mM NaCl, 5 mM EDTA, and 10% glycerol). 10% of the lysates were used as the protein inputs. The remnant lysates were centrifuged at 12,000 g for 10 min at 4°C, and the supernatants were prepared for immunoprecipitation overnight at 4°C using anti-HA antibody (Proteintech, 66006-2-Ig), anti-Flag antibody (Sigma, F1840), or anti-GFP antibody (Proteintech, 66002-1-Ig) with Protein-A/G Magnetic Beads (MCE). Immunoprecipitation beads were washed with lysis buffer five times. The washed immunoprecipitation beads were dissolved in lysis buffer for Western blot analysis.

### *In Vitro* pull-down assays

These experiments were performed as previously described [46]. In brief, the plasmids encoding GST and GST-2B were transformed into BL21 competent cells, which were induced with IPTG (1 mM) at 37°C for 3 h. Cells were then lysed in lysis buffer (20 mM Tris–HCl, 200 mM NaCl, 5% glycerol, and 0.3% Triton X-100) and the proteins were purified through affinity chromatography using a matrix (Transgen Biotech) followed by glutathione (10 mM in 50 mM Tris–HCl) elution and dialysis. HEK293T cells were transfected with plasmids encoding Flag-NLRP3 for 48 h and then were lysed with NP-40 lysis buffer, followed by immunoprecipitation with anti-Flag agarose (Sigma). Flag-NLRP3 was obtained by elution with 3xFLAG peptide (100 mg/ml in PBS) (Sigma). The purified GST or GST-2B (5 μg) were incubated with Flag-NLRP3 (5 μg) at 4°C overnight followed by glutathione agarose pull-down at room temperature for 2 h in PBS containing protease inhibitors. The glutathione agarose was washed three times with PBS and subject to immunoblot analysis.

### Cytotoxicity assay, Enzyme-linked immunosorbent assay (ELISA) and flow cytometry

Cell death was measured using culture supernatants by a lactate dehydrogenase (LDH) assay using LDH Cytotoxicity Assay Kit (Invitrogen) according to the manufacture’s instructions. The concentrations of IL-1β in culture supernatants or murine serum were measured via using the ELISA Kit (R&D Systems) according to the manufacture’s instructions. For flow cytometry, cells were incubated with Fluo-4 AM for 1 h at 37°C and washed five times, followed by resuspending in PBS containing 2% FBS, ready for detecting by BD LSRFortessa. The data were analyzed by FlowJo software.

### ASC oligomerization detection

Cells were lysed in ice-cold buffer (1% Triton X-100, 0.5% sodium deoxycholate, 50 mM Tris, pH 7.5, 150 mM NaCl, 5 mM EDTA, and 10% glycerol). The pellets were washed and then cross-linked with fresh DSS (4 mM) (Sigma-Aldrich, St. Louis, MO, USA) at 37°C for 30 min. The cross-linked samples were mixed with SDS loading buffer and boiled for 10 min for Western blot analysis.

### RNA isolation and quantitative RT-PCR

Total RNA was extracted with Cell Total RNA Isolation Kit (FOREGENE) following the manufacturer’s instructions. The first-strand complementary DNA (cDNA) was reverse-transcribed with PrimeScript 1st strand cDNA Synthesis kit (Takara) and gene expression was examined with Hieff qPCR SYBR Green Master (low rox) Mix (Yeasen) using ABI QuantStudio Q3 real-time PCR system. Data were normalized to the expression of the gene encoding β-actin. The sequences of the primers used in this study were as follows: ECHO 11-F: 5’CGGCCCCTGAATGCGGCTAA3’, ECHO 11-R: 5’ GAAACACGGACACCCAAAGTA3’; Mouse IL-1β-F: 5’CCTCTGATGGGCAACCACTT3’, Mouse IL-1β-R: 5’TTCATCCCCCACACGTTGAC3’; Mouse TNF-α-F: 5’ACTGAACTTCGGGGTGATCG3’ Mouse TNF-α-R: 5’TCTTTGAGATCCATGCCGTTG3’ Mouse IL-6-F: 5’GGGAAATCGTGGAAATGAGAAA3’ Mouse IL-6-R: 5’ATCCAGTTTGGTAGCATCCATC3’ Mouse β-actin-F: 5’- CACTGCCGCATCCTCTTCCTCCC-3’, Mouse β-actin-R:5’- CAATAGTGATGACCTGGCCGT-3’. Human IL-1β-F: 5’CTTCCTTCCTTCCTTCCT3’, Human IL-1β-R: 5’CAGAGCCTCATAGCAGTA3’; Human TNF-α-F: 5’GCCGCATCGCCGTCTCCTAC3’, Human TNF-α-R: 5’CCTCAGCCCCCTCTGGGGTC3’; Human IL-6-F: 5’AGACAGCCACTCACCTCTTCAG3’, Human IL-6-R: 5’TTCTGCCAGTGCCTCTTTGCTG3’; Human IL-8-F: 5’ACTGAGAGTGATTGAGAGTGGAC3’, Human IL-8-R: 5’AACCCTCTGCACCCAGTTTTC3’; Human IL-10-F: 5’TGTCCAGCTGGTCCTTTGTT3’, Human IL-10-R: 5’ACTGCACCCACTTCCCAGT3’; Human IL-17-F: 5’CAGGGAGAGCTTCATCTGTGT3’, Human IL-17-R: 5’GCTGAGCTTTGAGGGATGAT3’; Human IFN-α-F: 5’AGAATCTCTCCTTTCTCCTG3’, Human IFN-α-R: 5’TCTGACAACCTCCCAGGCAC3’; Human IFN-β-F: 5’TTGTTGAGAACCTCCTGGCT3’, Human IFN-β-R: 5’TGACTATGGTCCAGGCACAG3’; Human IFN-γ-F: 5’GACTGTGATTGCGGGGTTGT3’, Human IFN-γ-R: 5’GGCCCGGAGTGTAGACATCT3’; Human β-actin-F: 5’AGAGCTACGAGCTGCCTGAC3’, Human β-actin-R: 5’AGCACTGTGTTGGCGTACAG3’.

### Confocal microscopy

HEK293T and HeLa grown on sterile cover slips were transfected with indicated plasmids at 40% confluence for 24 h. PMA-differentiated THP-1-CT or THP-1-2B stable cells were grown on sterile cover slips for 24 h. Cells were washed three times with PBS, and fixed with 4% paraformaldehyde for 45 min. After washing three times with PBS, cells were permeabilized with 0.2% Triton X-100 for 20 min and washed three times with PBS. After that, cells were blocked with PBS containing 5% BSA for 1 h, and then incubated overnight with anti-HA antibody, anti-Flag antibody, or anti-NLRP3 antibody (1:50 in 5% BSA), respectively, followed by staining with Alexa Fluor 568-conjugated goat anti-rabbit IgG, Alexa Fluor 488-conjugated donkey anti-goat IgG (Invitrogen) (1:1000 in 5% BSA). Nuclei were stained with DAPI (Beyotime) for 5 min. Cells were viewed under confocal fluorescence microscope (Nikon).

### Animal study

To construct AAV2/9-EGFP-2B-Flag, AAV2/9-EGFP-2B_I65S/V67S_-Flag and AAV2/9-EGFP, 2B and 2B_I65S/V67S_ ORF was cloned into pcAAV-CAG-EGFP-3xFlag-tWPA vector, named pcAAV-CAG-EGFP-2B-3xFlag-tWPA and pcAAV-CAG-EGFP-2B_I65S/V67S_ -3xFlag-tWPA, and then transfected into 293T cells with AAV helper plasmids. Cells were collected 96h after transfection, and the viral particles were released from cells by freeze thaw cycling and sonication. The virus was purified using cesium chloride density-gradient ultracentrifugation and dialyzed into PBS buffer.

Six-week-old C57BL/6 mice purchased from the animal housing facility of the Chinese Academy of Sciences (Changsha, China). Mice were tail vein-injected with 200 μL containing 4 × 10^11^ VG (Vector Genomes) of AAV2/9-EGFP-2B-Flag, AAV2/9-EGFP-2B_I65S/V67S_-Flag or AAV2/9-EGFP for 2 weeks. And then treated with MCC950 (10 mg/kg in PBS) or Ac-YVAD-cmk (8 mg/kg, dissolved in 1% vol/vol DMSO in PBS) every 2 days by intraperitoneal injection for AAV2/9-EGFP-2B-Flag or AAV2/9-EGFP mice for a week. Mouse tissues were collected for Western blotting, immunofluorescence and immunohistochemistry analysis.

For immunohistochemistry (IHC), the sections were deparaffinized and rehydrated in xylene and ethanol. Endogenous peroxidase was quenched by incubation in 3% hydrogen peroxide, and antigen retrieval was performed in 0.01 M citrate buffer. Sections were then blocked and incubated with primary rabbit monoclonal anti-cleaved N-terminal GSDMD (ab215203) (1:1000) and overnight at 4°C. After a washing step, biotinylated secondary antibodies were applied. The avidin-biotin-peroxidase complex (VectaStain standard ABC kit; Vector Laboratories, Burlingame, CA) was used to localize the biotinylated antibody. Diaminobenzidine (DAB; Vector Laboratories) was utilized for color development. Negative control was performed by substituting primary antibodies with PBS. The integrated optical density of DAB signals was determined by using an Image-Pro Plus (Media Cybernetics, Bethesda, MD).

All experiments were performed according to protocols approved by the Institutional Animal Care and Use Committee of Wuhan Institute of Virology.

### Statistical analyses

Statistical analysis was conducted via using the *t*-test for two groups and one-way ANOVA for multiple groups (GraphPad Prism 8). Date was considered statistically significant when *P < 0.05.

## Supporting information

S1 Fig ECHO 11 induces IL-1β secretion and LDH release in Huh7 and HepG2 cells. (A-B) The mRNA levels of the immune and pro-inflammatory genes in ECHO 11-infected Huh7 and HepG2 cells, as indicated, were measured by qRT-PCR at 24 h.p.i., and the level of each target gene in uninfected cells was defined as 1-fold. Data represent means and SD from three repeated experiments. ****, *P* < 0.0001, as measured by unpaired *t* test.
(TIF)

S2 Fig The RNA replication and 2B protein of ECHO 11 in THP-1 cells and BMDMs. THP-1 cells (**A**) and BMDMs (**C**) were infected with ECHO 11 at an MOI of 0.1, 0.5 and 1 for 24 h, or THP-1 cells (**B**) and BMDMs (**D**) were infected with 0.1 MOI ECHO 11 for 12, 24 and 48 h. Total RNAs were extracted and the accumulation of viral RNA was measured via qRT-PCR, and the level of viral RNA in uninfected cells (mock) was defined as 1-fold. Cell lysates were analyzed by immunoblotting using antibodies specific for ECHO 11 2B. Data represent means and SD from three repeated experiments.
(TIF)

S3 Fig ECHO 11 induces the production of TNF-α and IL-6 in THP-1 cells and BMDMs. THP-1 cells were treated with LPS/Nigericin or infected with ECHO 11 at an MOI of 0.1, 0.5 and 1 for 24 h (**A-B**) or infected with 0.1 MOI ECHO 11 for 12, 24 and 36 h (**C-D**). BMDMs were treated with LPS/Nigericin or infected with ECHO 11 at an MOI of 0.5 for 24 h (**E-F**) or infected with 0.1 MOI ECHO 11 for 24 h (G-H). The mRNA levels of TNF-α (A, C, E and G) and IL-6 (B, D, F and H) were determined by qRT-PCR, and the level of each target gene in untreated cells (mock) was defined as 1-fold. Data represent means and SD from three repeated experiments. *, *P* < 0.05; **, *P* < 0.01; ***, *P* < 0.001; ****, *P* < 0.0001; ns, not significant, as measured by one-way ANOVA.
(TIF)

S4 Fig VX-765 shows no effect on the RNA replication of ECHO 11 in THP-1 cells. THP-1 cells were infected with ECHO 11 at an MOI of 0.5 for 24 h in the presence of Casp-1 inhibitor VX-765 (50 μM). The accumulation of viral RNA was measured with qRT-PCR, and the level of viral RNA in infected cells treated with vehicle was defined as 1-fold. Data represent means and SD from three repeated experiments. ns, not significant, as measured by unpaired *t* test.
(TIF)

S5 Fig The RNA replication of ECHO 11 in THP-1 cells knocking down the different components of NLRP3 inflammasome. THP-1 stably expressing non-targeting shRNA (ctrl) or specific shRNA targeting NLRP3, ASC, CASP-1 and GSDMD were infected with ECHO 11 at an MOI of 0.5 for 24 h. The accumulation of viral RNA was measured with qRT-PCR, and the level of viral RNA in cells expressing shCtrl was defined as 1-fold. Data represent means and SD from three repeated experiments. ns, not significant, as measured by one-way ANOVA.
(TIF)

S6 Fig ECHO 11 infection induces the activation of NLRP3-inflammasomes. (**A-B**) THP-1 cells stably expressing a second set of siRNAs targeting the key components of NLRP3 inflammasome, which was different from that was used in Fig 3D and 3E, were treated with LPS /Nigericin (A) or infected with ECHO 11 at an MOI of 0.5 for 24 h (B). GSDMD-N, P17 and P20 in the supernatants, and GSDMD, pro-IL-1β and Casp-1 in the lysates were examined by Western blotting.
(TIF)

S7 Fig The replication of ECHO 11 is required for NLRP3 inflammasome activation. (**A-J**) THP-1 cells were infected with UV- or heat-inactivated or live ECHO 11 at an MOI of 0.5 for 24 h. (**A, F**) GSDMD-N, P17 and P20 in the supernatants, and GSDMD, pro-IL-1β and CASP-1 in the lysates were examined via Western blotting. (**B, G**) The LDH release in the supernatants were determined by cytotoxicity assay, and the level of LDH in untreated cells (mock) was defined as 1-fold. (**C, H**) Mature IL-1β levels in supernatants were determined by ELISA. (**D, I**) The intracellular mRNA levels of IL-1β were measured by qRT-PCR, and the level of IL-1β mRNA in mock cells were defined as 1-fold. (**E, J**) The accumulation of viral RNA was measured with qRT-PCR, and the level of viral RNA in uninfected cells (mock) was defined as 1-fold. (**K-O**) THP-1 cells were transfected with two different siRNAs targeting ECHO 11 genomic sequences and infected with ECHO 11 at an MOI of 0.5 for 24 h, GSDMD-N, P17 and P20 (K), the LDH release in the supernatants (L), mature IL-1β levels in supernatants (M), the intracellular mRNA levels of IL-1β (N), the accumulation of viral RNA (O) were determined. Data represent means and SD from three repeated experiments. *, *P* < 0.05; **, *P* < 0.01; ***, *P* < 0.001; ****, *P* < 0.0001; ns, not significant, as measured by one-way ANOVA.
(TIF)

S8 Fig ECHO 11 2B interacts with NLRP3. (**A**) 293T cells were co-transfected with plasmids encoding HA-2B and GFP-NLRP3, the subcellular localizations of HA-2B (red), GFP-NLRP3 (green) and nucleus marker DAPI (blue) were analyzed with confocal microscopy. Scale bar, 10 μm. (**B**) 293T cells were co-transfected with plasmids encoding HA-2B and Flag-NLRP6, subjected to Co-IP using anti-Flag antibody or anti-IgG antibody, and analyzed by Western blotting with anti-Flag and anti-HA antibodies.
(TIF)

S9 Fig Activation of NLRP3 inflammasome by ECHO 11 2B is independent on Ca^2+^ flux. (**A-B**) THP-1 cells were infected with control (CT), ECHO 11 2B-encoding or EMCV 2B-encoding lentivirus, and then differentiated into macrophages with PMA. The 2B-expressing THP-1 macrophages were then treated with BAPTA-AM at doses of 0, 5, 10, 20, and 40 μM. The resulting cells were incubated with Fluo-4 AM and the levels of Ca^2+^ in different groups were analyzed by flow cytometry and quantified with FlowJo software. (**C**) GSDMD-N, P17 and P20 in the supernatants and GSDMD, pro-IL-1β, Casp-1, ECHO 11 2B and EMCV 2B in the lysates were determined by western blot. (**D**) Mature IL-1β levels in the supernatants of 2B stably expressing THP-1 cells were determined by ELISA. (**E**) The LDH release in the supernatants were determined by cytotoxicity assay, and the level of LDH in the controlled THP-1 cells was defined as 1-fold. Data represent means and SD from three repeated experiments. ***, *P* < 0.001; ****, *P* < 0.0001; ns, not significant, as measured by one-way ANOVA.
(TIF)

S10 Fig ECHO 11 2B induces NLRP3 inflammasome activation in murine liver. **(A-D)** C57BL/6 mice were tail vein-injected 4 × 10^11^ VG of AAV2/9-EGFP-2B-Flag (AAV-2B) (n = 3) or AAV2/9-EGFP (AAV-CT) (n = 3) for 2 weeks, and then treated with the vehicle, MCC950 (10 mg/kg) or Ac-YVAD-cmk (8 mg/kg) every 2 days via i.p. injection for a week. At that, mice were euthanized and the livers were collected. (**A**) Immunofluorescence analysis of EGFP and EGFP-fused 2B (green), and DAPI (blue) in the liver tissue sections. Scale bar is 100 μm (10×) or 20 μm (60×). (**B**) The fluorescence intensity of AAV-EGFP in each group was quantified using Image J software. Data represent means and SD, **, *P* < 0.01; ***, *P* < 0.001; ****, *P* < 0.0001; ns, not significant, as measured by one-way ANOVA. (**C-D**) GSDMD-N, P17, GSDMD, and pro-IL-1β in the livers of different groups were analyzed by Western blotting.
(TIF)

S11 Fig ECHO 11 2B activates NLRP3 inflammasome in murine spleen. (**A**) Immunohistochemistry analysis of N-GSDMD in murine spleen of different groups as indicated in Fig 7. Scale bar is 200 μm (10×) or 50 μm (60×). (**B**) The relative expression of N-GSDMD was quantified using Image J software, and the level of N-GSDMD in mock group was defined as 1-fold. ***, *P* < 0.001; ****, *P* < 0.0001; ns, not significant, as measured by one-way ANOVA.
(TIF)

S12 Fig The 2Bs of multiple EVs interact with NLRP3 and induce NLRP3 inflammasome activation. (**A**) 293T cells were co-transfected with plasmids encoding NLRP3, ASC, Caspase-1, GSDMD and pro-IL-1β, together with the empty vector or 2B proteins of ECHO 11, EV71, CVA16, or CVB3. GSDMD-N, P20 or P17 in the supernatants, and GSDMD, NLRP3, ASC, CASP-1, 2B and pro-IL-1β in the lysates were examined by Western blotting. (**B**) The LDH release in the supernatants were determined by cytotoxicity assay, and the level of LDH in cells transfected with the empty vector was defined as 1-fold. (**C**) Mature IL-1β levels in supernatants were determined by ELISA. ***, *P* < 0.001; ****, *P* < 0.0001, as measured by one-way ANOVA. (**D**) 293T cells were co-transfected with plasmids encoding Flag-NLRP3 and HA-tagged 2Bs of ECHO 11, EV71, CVA16 or CVB3), subjected to Co-IP using anti-Flag antibody, and analyzed by Western blotting with anti-Flag and anti-HA antibodies.
(TIF)
