# Supplementary figures and images for: Echovirus 11 infection induces pyroptotic cell death by facilitating NLRP3 inflammasome activation
Source: PLoS Pathog. 2022 Aug 26;18(8):e1010787. doi: 10.1371/journal.ppat.1010787 (PMC9455886; doi:10.1371/journal.ppat.1010787)

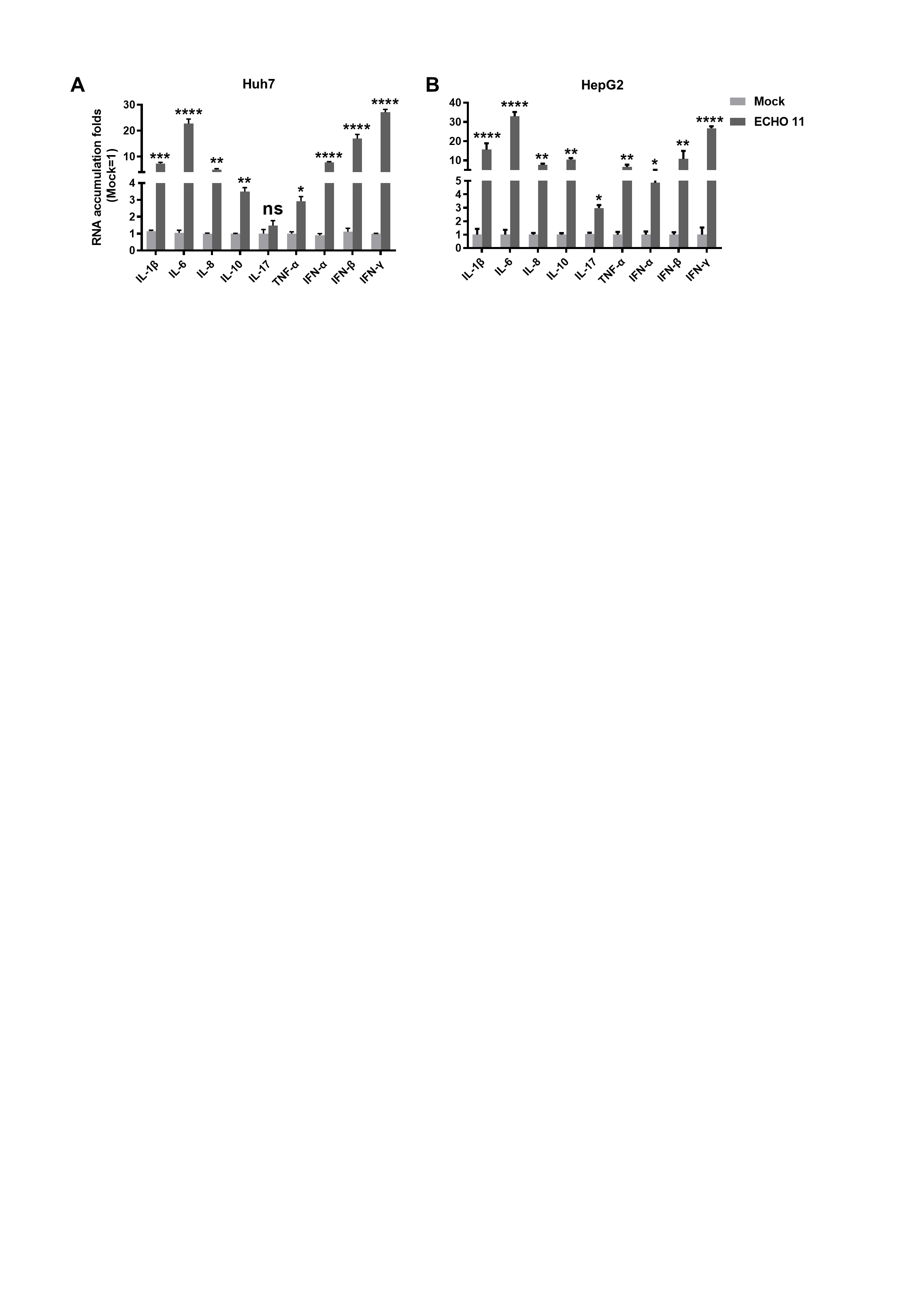

Supplement: S1 Fig — (A-B) The mRNA levels of the immune and pro-inflammatory genes in ECHO 11-infected Huh7 and HepG2 cells, as indicated, were measured by qRT-PCR at 24 h.p.i., and the level of each target gene in uninfected cells was defined as 1-fold. Data represent means and SD from three repeated experiments. ****, P < 0.0001, as measured by unpaired t test. (TIF) [file ppat.1010787.s001.tif]

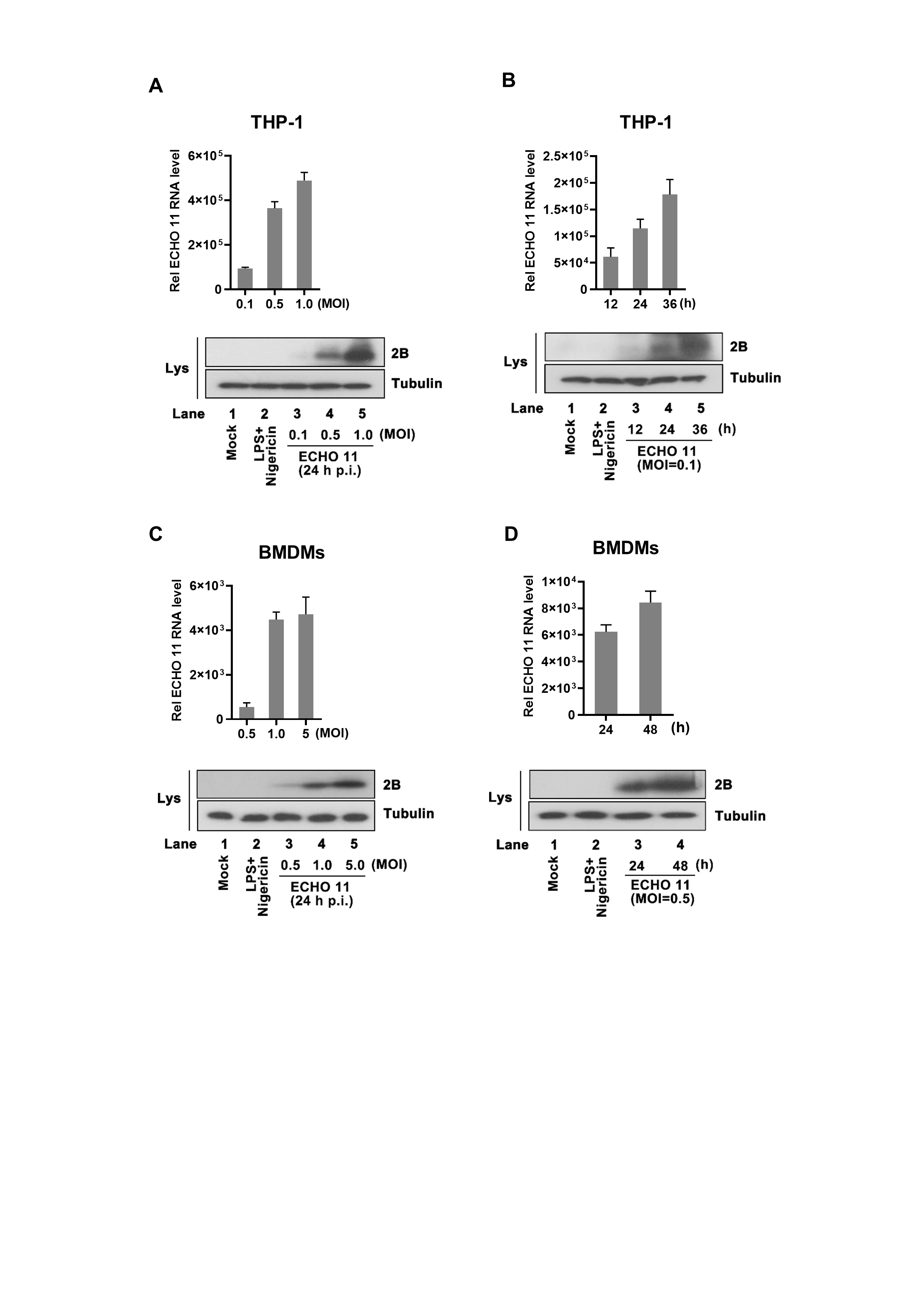

Supplement: S2 Fig — THP-1 cells (A) and BMDMs (C) were infected with ECHO 11 at an MOI of 0.1, 0.5 and 1 for 24 h, or THP-1 cells (B) and BMDMs (D) were infected with 0.1 MOI ECHO 11 for 12, 24 and 48 h. Total RNAs were extracted and the accumulation of viral RNA was measured via qRT-PCR, and the level of viral RNA in uninfected cells (mock) was defined as 1-fold. Cell lysates were analyzed by immunoblotting using antibodies specific for ECHO 11 2B. Data represent means and SD from three repeated experiments. (TIF) [file ppat.1010787.s002.tif]

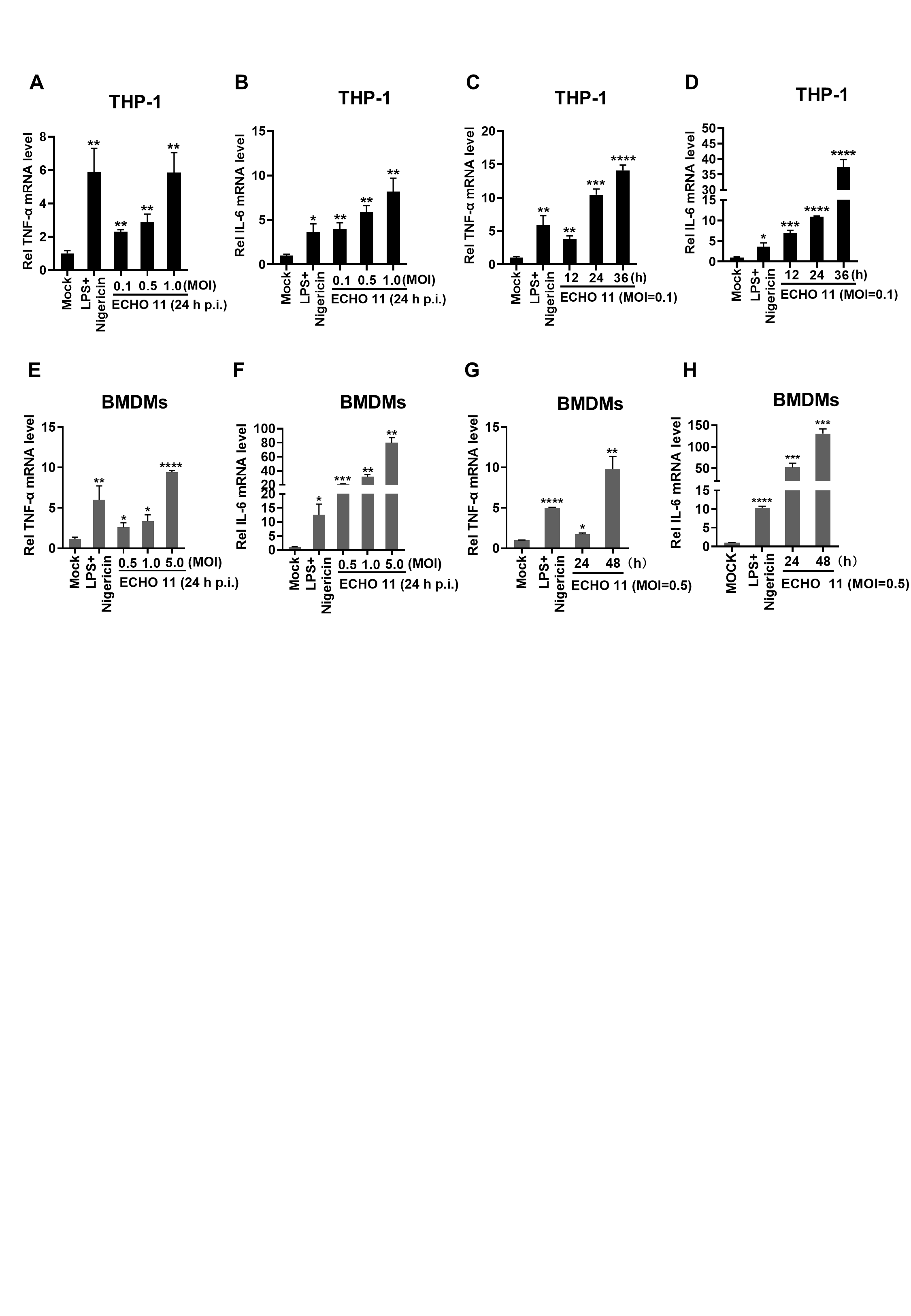

Supplement: S3 Fig — THP-1 cells were treated with LPS/Nigericin or infected with ECHO 11 at an MOI of 0.1, 0.5 and 1 for 24 h (A-B) or infected with 0.1 MOI ECHO 11 for 12, 24 and 36 h (C-D). BMDMs were treated with LPS/Nigericin or infected with ECHO 11 at an MOI of 0.5 for 24 h (E-F) or infected with 0.1 MOI ECHO 11 for 24 h (G-H). The mRNA levels of TNF-α (A, C, E and G) and IL-6 (B, D, F and H) were determined by qRT-PCR, and the level of each target gene in untreated cells (mock) was defined as 1-fold. Data represent means and SD from three repeated experiments. *, P < 0.05; **, P < 0.01; ***, P < 0.001; ****, P < 0.0001; ns, not significant, as measured by one-way ANOVA. (TIF) [file ppat.1010787.s003.tif]

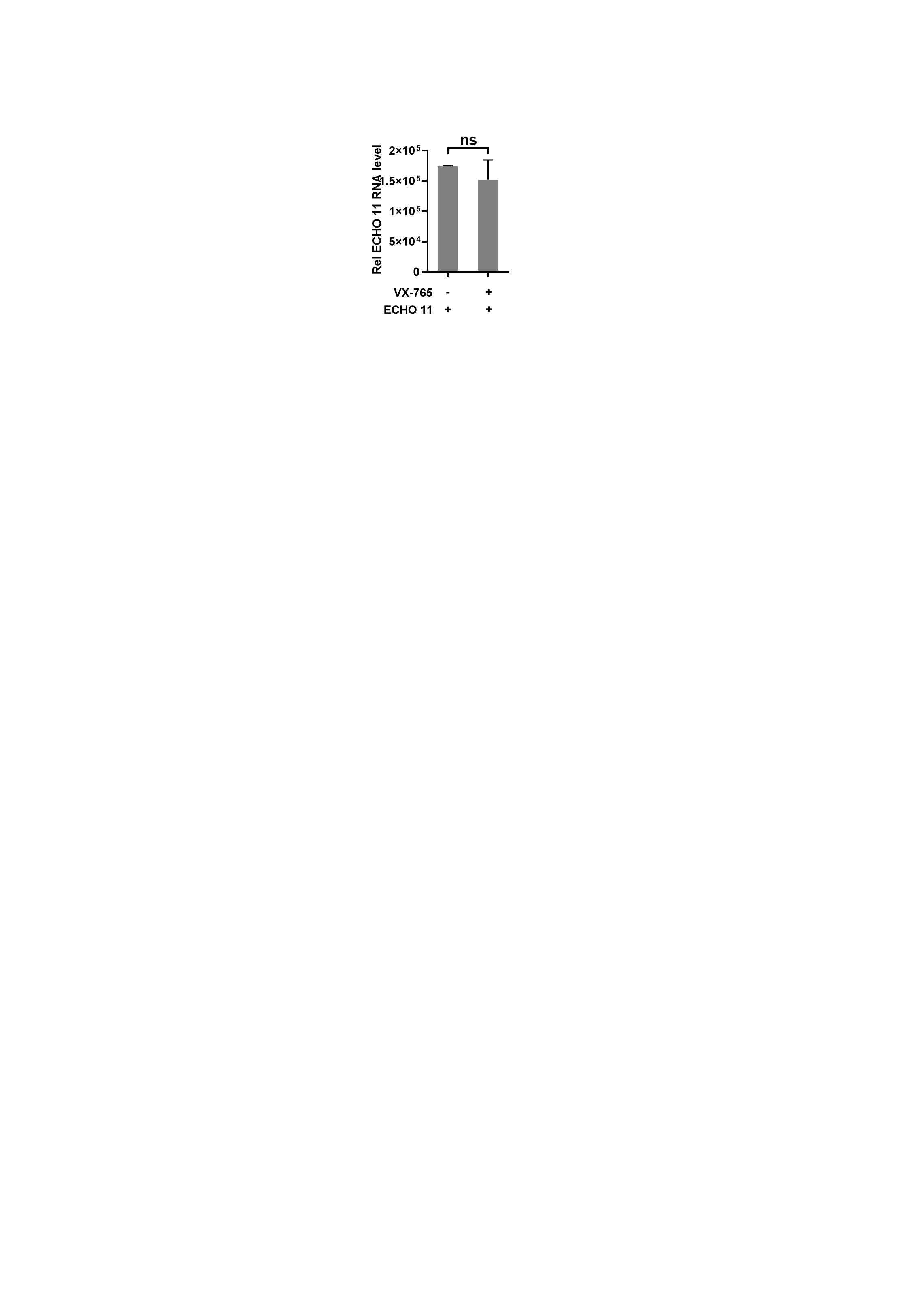

Supplement: S4 Fig — THP-1 cells were infected with ECHO 11 at an MOI of 0.5 for 24 h in the presence of Casp-1 inhibitor VX-765 (50 μM). The accumulation of viral RNA was measured with qRT-PCR, and the level of viral RNA in infected cells treated with vehicle was defined as 1-fold. Data represent means and SD from three repeated experiments. ns, not significant, as measured by unpaired t test. (TIF) [file ppat.1010787.s004.tif]

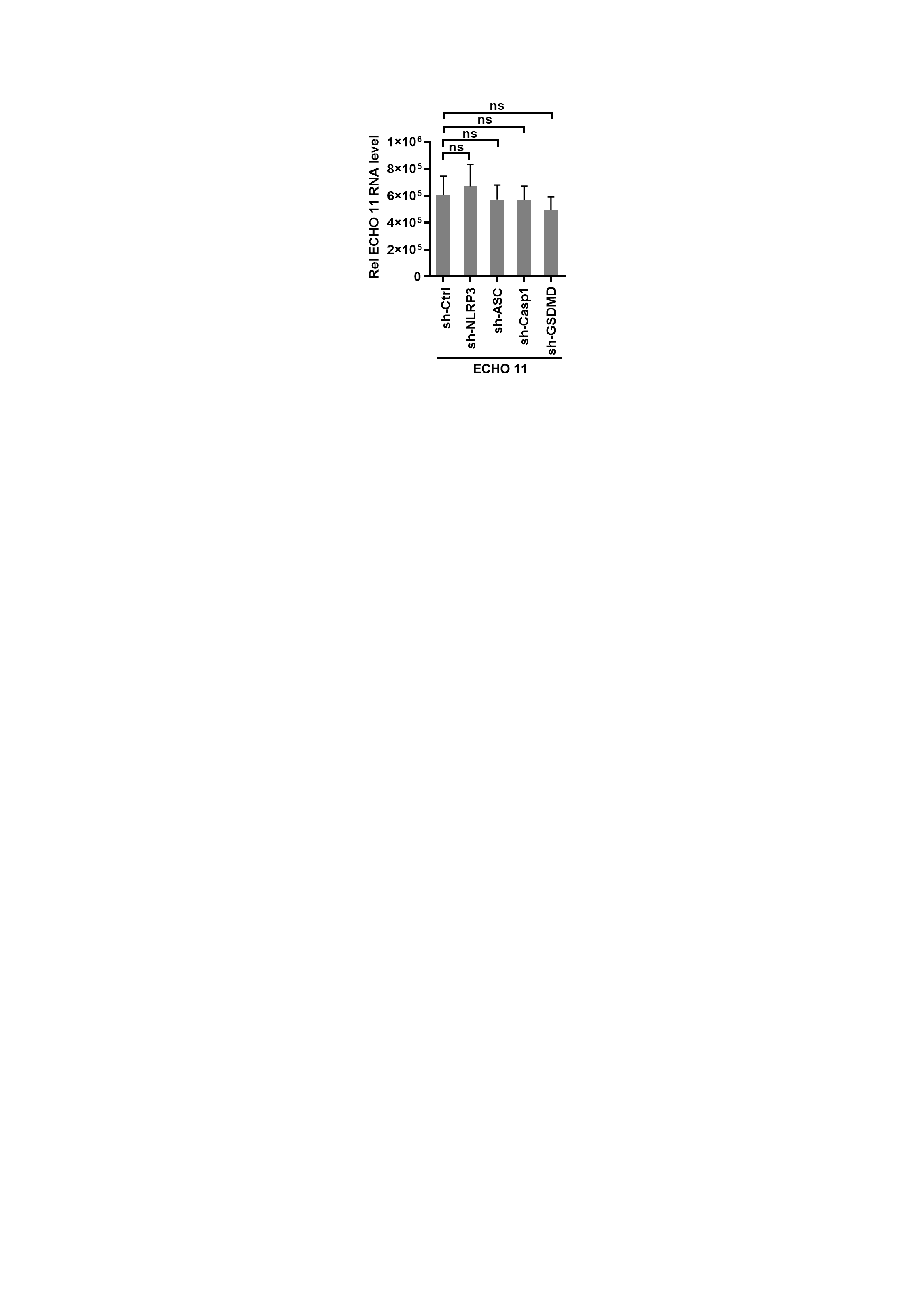

Supplement: S5 Fig — THP-1 stably expressing non-targeting shRNA (ctrl) or specific shRNA targeting NLRP3, ASC, CASP-1 and GSDMD were infected with ECHO 11 at an MOI of 0.5 for 24 h. The accumulation of viral RNA was measured with qRT-PCR, and the level of viral RNA in cells expressing shCtrl was defined as 1-fold. Data represent means and SD from three repeated experiments. ns, not significant, as measured by one-way ANOVA. (TIF) [file ppat.1010787.s005.tif]

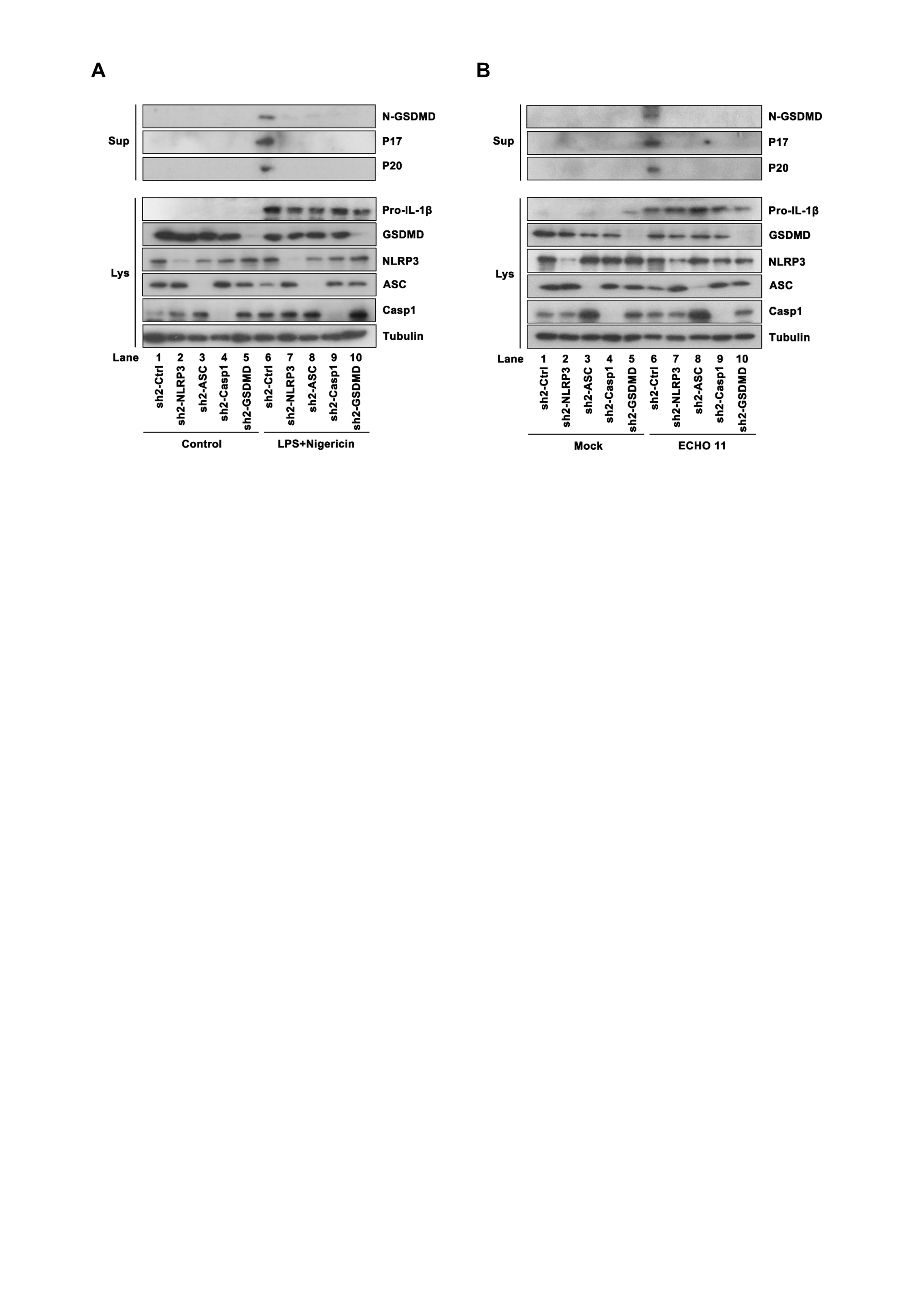

Supplement: S6 Fig — (A-B) THP-1 cells stably expressing a second set of siRNAs targeting the key components of NLRP3 inflammasome, which was different from that was used in Fig 3D and 3E, were treated with LPS /Nigericin (A) or infected with ECHO 11 at an MOI of 0.5 for 24 h (B). GSDMD-N, P17 and P20 in the supernatants, and GSDMD, pro-IL-1β and Casp-1 in the lysates were examined by Western blotting. (TIF) [file ppat.1010787.s006.tif]

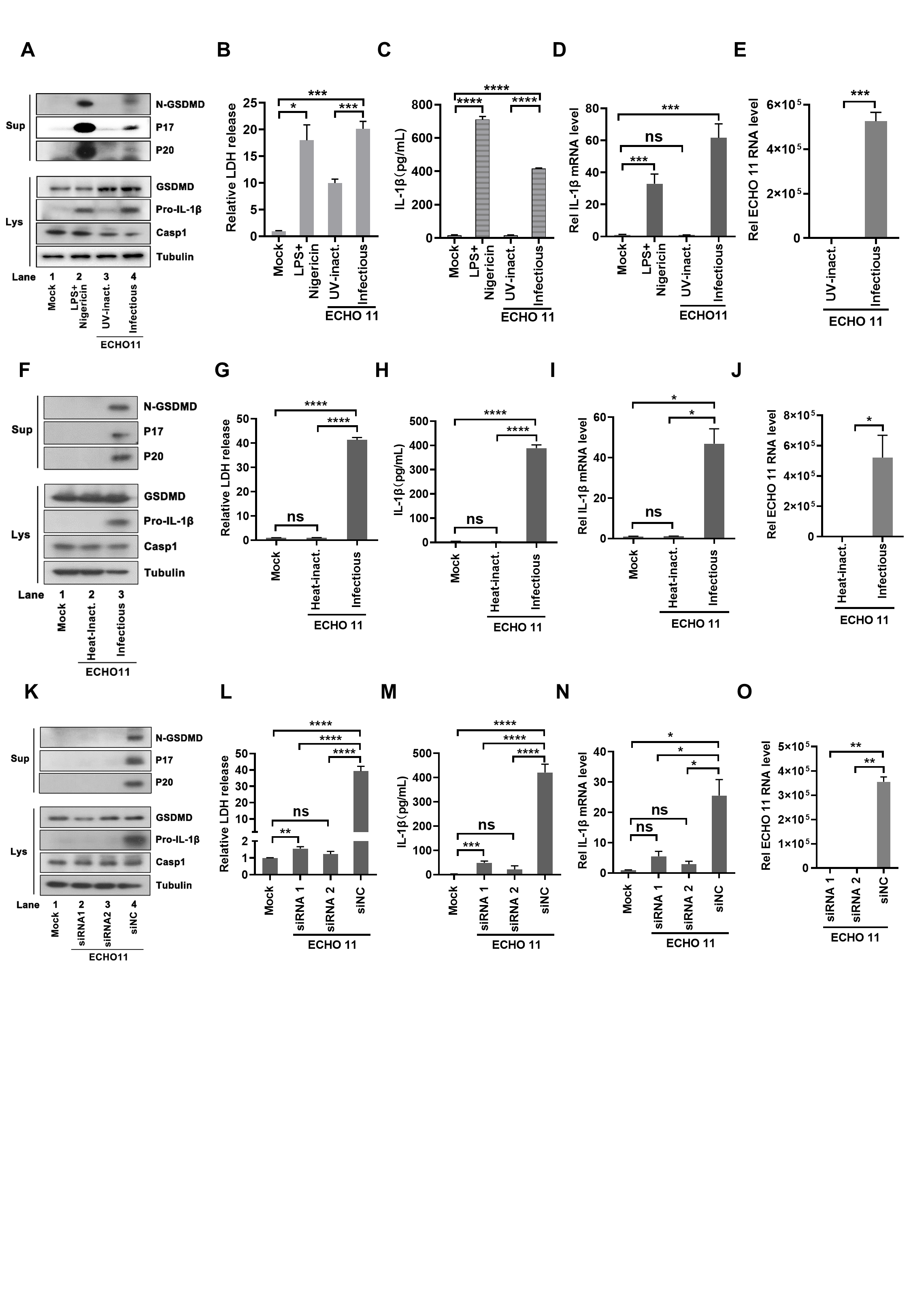

Supplement: S7 Fig — (A-J) THP-1 cells were infected with UV- or heat-inactivated or live ECHO 11 at an MOI of 0.5 for 24 h. (A, F) GSDMD-N, P17 and P20 in the supernatants, and GSDMD, pro-IL-1β and CASP-1 in the lysates were examined via Western blotting. (B, G) The LDH release in the supernatants were determined by cytotoxicity assay, and the level of LDH in untreated cells (mock) was defined as 1-fold. (C, H) Mature IL-1β levels in supernatants were determined by ELISA. (D, I) The intracellular mRNA levels of IL-1β were measured by qRT-PCR, and the level of IL-1β mRNA in mock cells were defined as 1-fold. (E, J) The accumulation of viral RNA was measured with qRT-PCR, and the level of viral RNA in uninfected cells (mock) was defined as 1-fold. (K-O) THP-1 cells were transfected with two different siRNAs targeting ECHO 11 genomic sequences and infected with ECHO 11 at an MOI of 0.5 for 24 h, GSDMD-N, P17 and P20 (K), the LDH release in the supernatants (L), mature IL-1β levels in supernatants (M), the intracellular mRNA levels of IL-1β (N), the accumulation of viral RNA (O) were determined. Data represent means and SD from three repeated experiments. *, P < 0.05; **, P < 0.01; ***, P < 0.001; ****, P < 0.0001; ns, not significant, as measured by one-way ANOVA. (TIF) [file ppat.1010787.s007.tif]

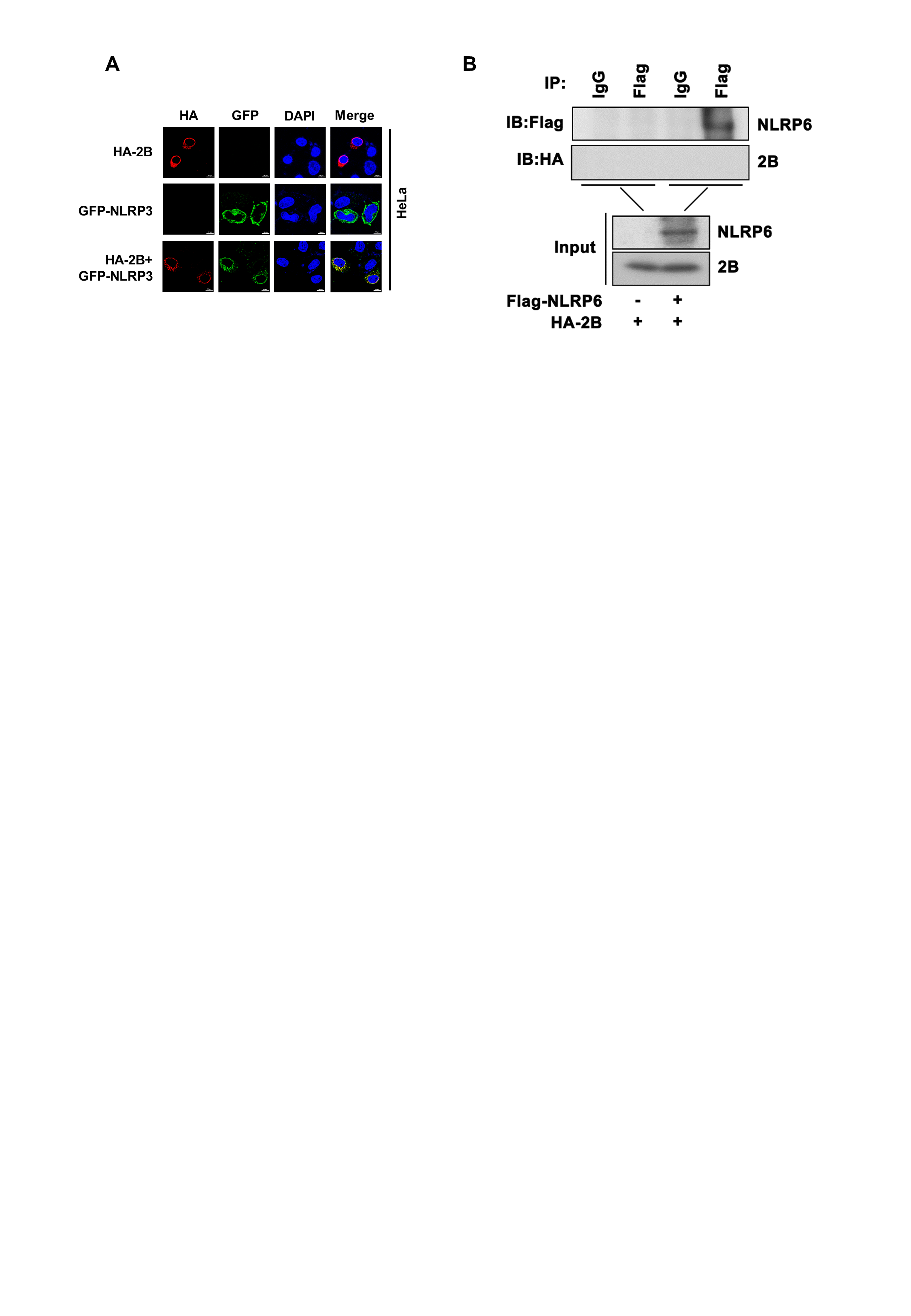

Supplement: S8 Fig — (A) 293T cells were co-transfected with plasmids encoding HA-2B and GFP-NLRP3, the subcellular localizations of HA-2B (red), GFP-NLRP3 (green) and nucleus marker DAPI (blue) were analyzed with confocal microscopy. Scale bar, 10 μm. (B) 293T cells were co-transfected with plasmids encoding HA-2B and Flag-NLRP6, subjected to Co-IP using anti-Flag antibody or anti-IgG antibody, and analyzed by Western blotting with anti-Flag and anti-HA antibodies. (TIF) [file ppat.1010787.s008.tif]

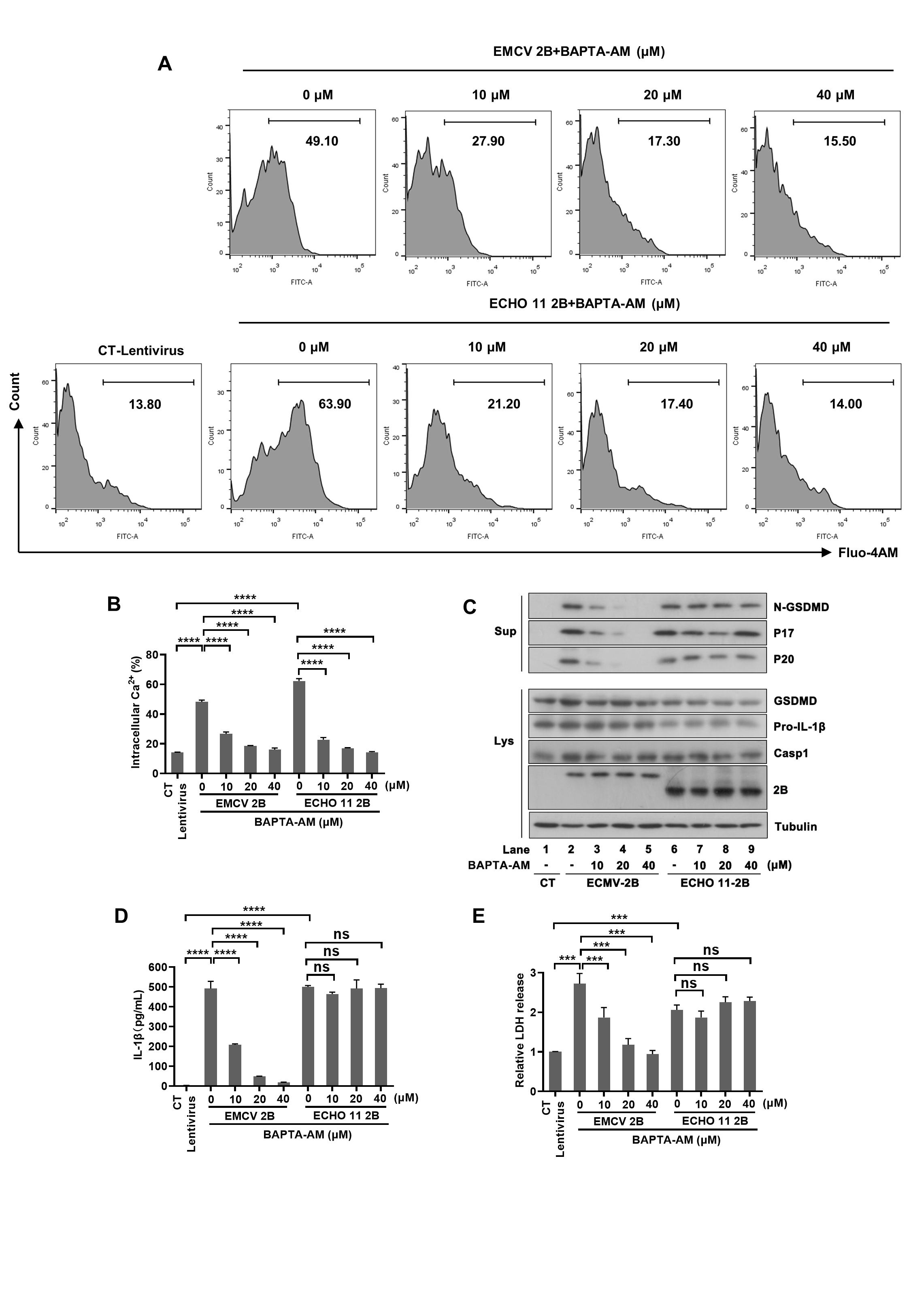

Supplement: S9 Fig — (A-B) THP-1 cells were infected with control (CT), ECHO 11 2B-encoding or EMCV 2B-encoding lentivirus, and then differentiated into macrophages with PMA. The 2B-expressing THP-1 macrophages were then treated with BAPTA-AM at doses of 0, 5, 10, 20, and 40 μM. The resulting cells were incubated with Fluo-4 AM and the levels of Ca2+ in different groups were analyzed by flow cytometry and quantified with FlowJo software. (C) GSDMD-N, P17 and P20 in the supernatants and GSDMD, pro-IL-1β, Casp-1, ECHO 11 2B and EMCV 2B in the lysates were determined by western blot. (D) Mature IL-1β levels in the supernatants of 2B stably expressing THP-1 cells were determined by ELISA. (E) The LDH release in the supernatants were determined by cytotoxicity assay, and the level of LDH in the controlled THP-1 cells was defined as 1-fold. Data represent means and SD from three repeated experiments. ***, P < 0.001; ****, P < 0.0001; ns, not significant, as measured by one-way ANOVA. (TIF) [file ppat.1010787.s009.tif]

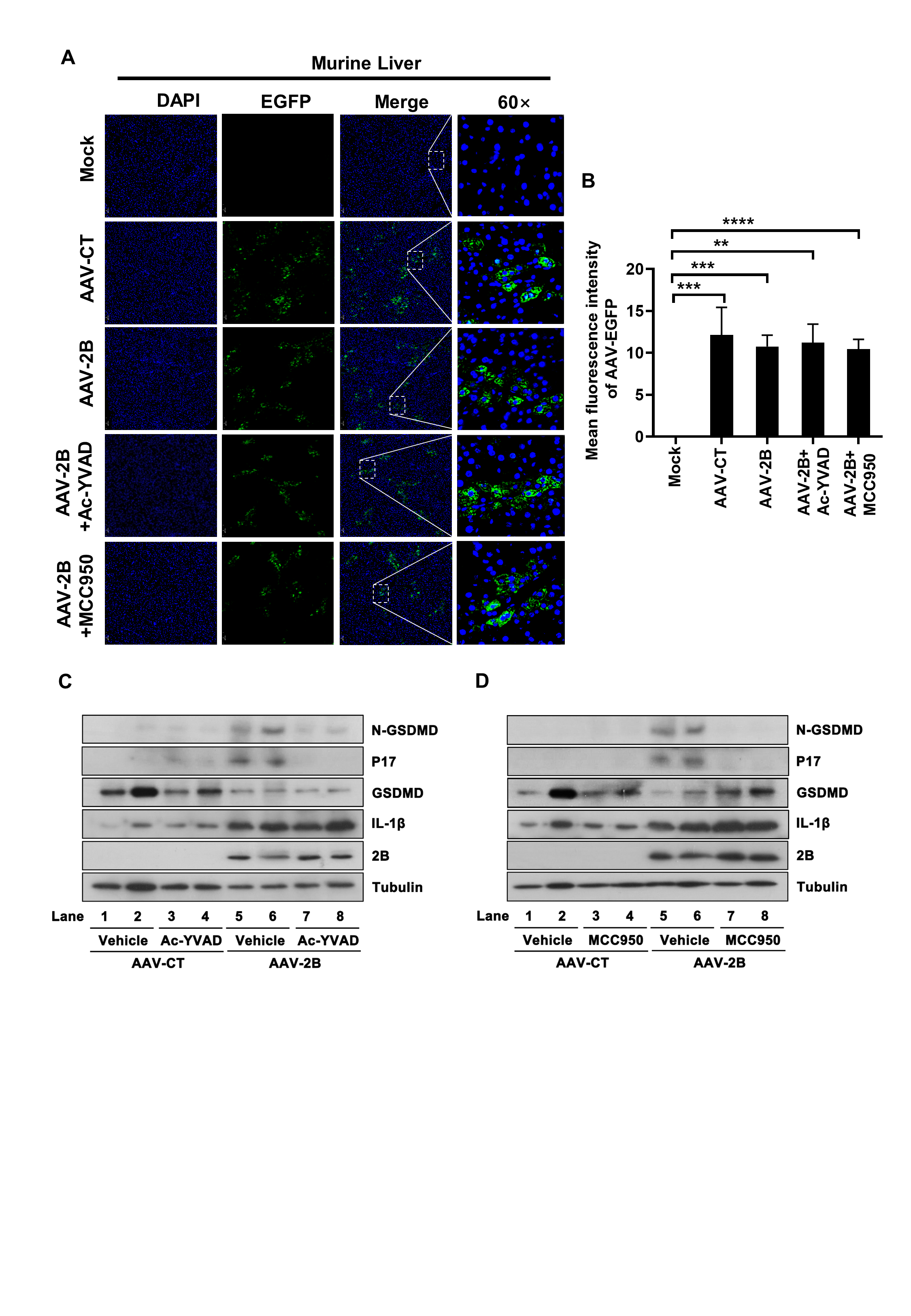

Supplement: S10 Fig — (A-D) C57BL/6 mice were tail vein-injected 4 × 10^11^ VG of AAV2/9-EGFP-2B-Flag (AAV-2B) (n = 3) or AAV2/9-EGFP (AAV-CT) (n = 3) for 2 weeks, and then treated with the vehicle, MCC950 (10 mg/kg) or Ac-YVAD-cmk (8 mg/kg) every 2 days via i.p. injection for a week. At that, mice were euthanized and the livers were collected. (A) Immunofluorescence analysis of EGFP and EGFP-fused 2B (green), and DAPI (blue) in the liver tissue sections. Scale bar is 100 μm (10×) or 20 μm (60×). (B) The fluorescence intensity of AAV-EGFP in each group was quantified using Image J software. Data represent means and SD, **, P < 0.01; ***, P < 0.001; ****, P < 0.0001; ns, not significant, as measured by one-way ANOVA. (C-D) GSDMD-N, P17, GSDMD, and pro-IL-1β in the livers of different groups were analyzed by Western blotting. (TIF) [file ppat.1010787.s010.tif]

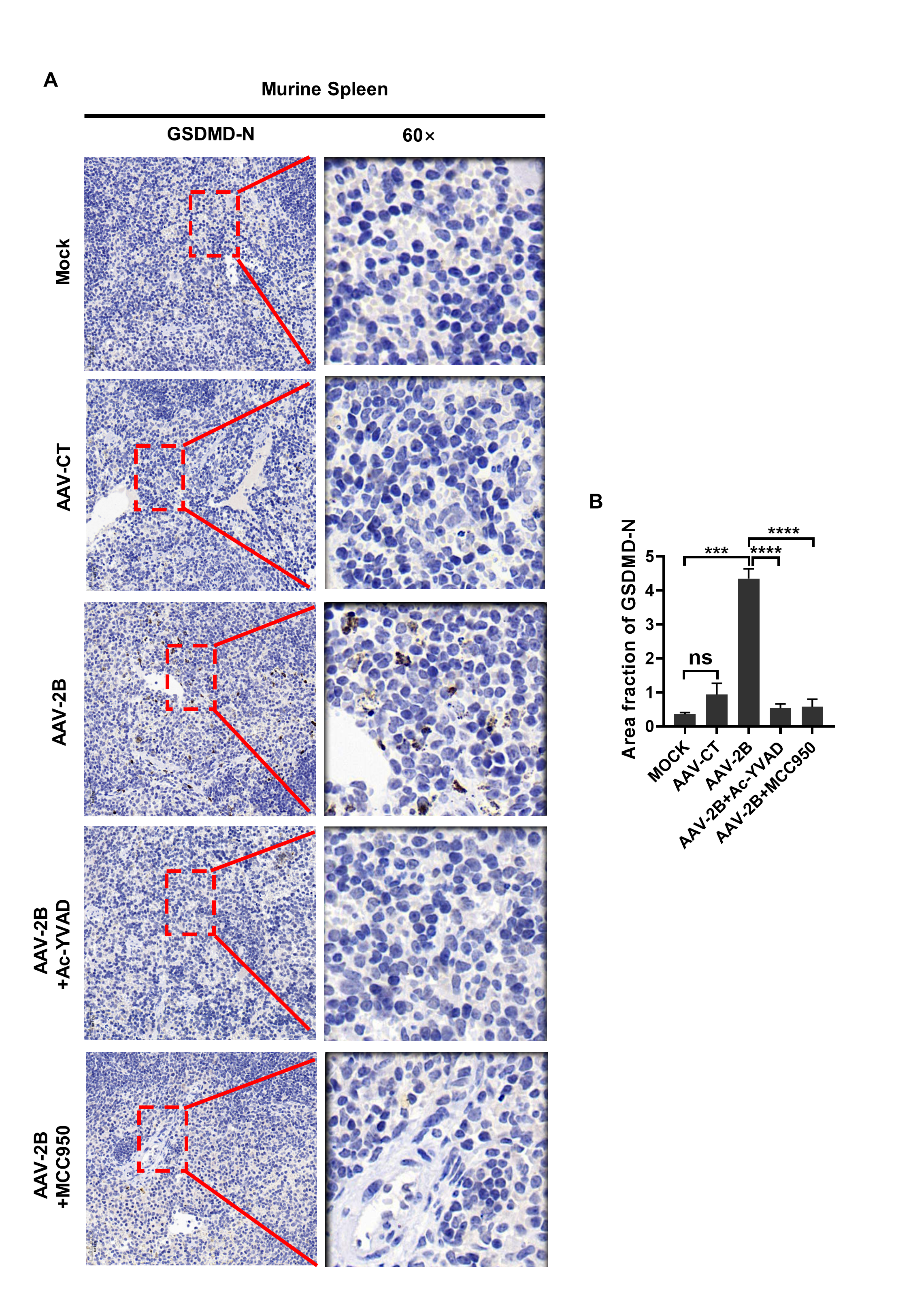

Supplement: S11 Fig — (A) Immunohistochemistry analysis of N-GSDMD in murine spleen of different groups as indicated in Fig 7. Scale bar is 200 μm (10×) or 50 μm (60×). (B) The relative expression of N-GSDMD was quantified using Image J software, and the level of N-GSDMD in mock group was defined as 1-fold. ***, P < 0.001; ****, P < 0.0001; ns, not significant, as measured by one-way ANOVA. (TIF) [file ppat.1010787.s011.tif]

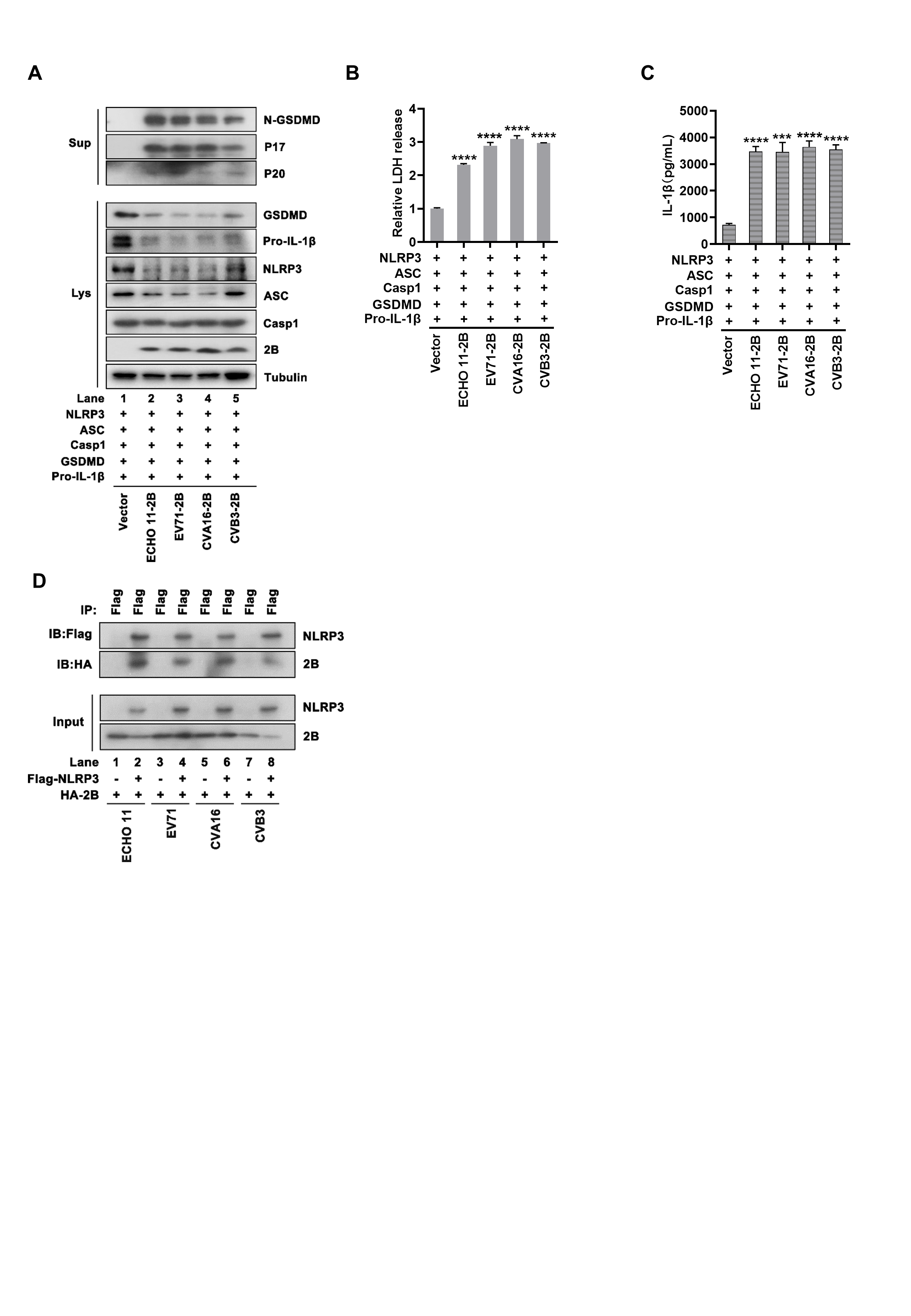

Supplement: S12 Fig — (A) 293T cells were co-transfected with plasmids encoding NLRP3, ASC, Caspase-1, GSDMD and pro-IL-1β, together with the empty vector or 2B proteins of ECHO 11, EV71, CVA16, or CVB3. GSDMD-N, P20 or P17 in the supernatants, and GSDMD, NLRP3, ASC, CASP-1, 2B and pro-IL-1β in the lysates were examined by Western blotting. (B) The LDH release in the supernatants were determined by cytotoxicity assay, and the level of LDH in cells transfected with the empty vector was defined as 1-fold. (C) Mature IL-1β levels in supernatants were determined by ELISA. ***, P < 0.001; ****, P < 0.0001, as measured by one-way ANOVA. (D) 293T cells were co-transfected with plasmids encoding Flag-NLRP3 and HA-tagged 2Bs of ECHO 11, EV71, CVA16 or CVB3), subjected to Co-IP using anti-Flag antibody, and analyzed by Western blotting with anti-Flag and anti-HA antibodies. (TIF) [file ppat.1010787.s012.tif]
